# Study on development methods of different types of gas wells in tight sandstone gas reservoirs

**DOI:** 10.1038/s41598-023-43640-7

**Published:** 2023-09-29

**Authors:** Jie He, Zhiwei Liu, Heng Zhang, Shenghong Xie, Xiqiang Wang, Yushuang Zhu

**Affiliations:** 1 State Key Laboratory of Continental Dynamics, Department of Geology, Northwest University, Xi’an, 710069 China; 2 No. 7 Oil Production Plant, PetroChina Changqing Oilfield Company, Xi’an, 710018 Shanxi China

**Keywords:** Fossil fuels, Engineering

## Abstract

Reasonable production allocation of tight sandstone gas reservoirs is an important basis for efficient development of gas wells. Taking Block XX in Ordos Basin as an example, the modified flowing material balance equation was established considering the variation of gas viscosity and compression coefficient, the advantages and disadvantages of the method were discussed, and a reasonable production allocation process for gas wells was developed. The results show that: ① The commonly used flow material balance method ignores the change of natural gas compression coefficient, viscosity and deviation coefficient in the production process. The slope of the relationship curve between bottom hole pressure and cumulative production and the slope of the relationship curve between average formation pressure and cumulative production are not equal After considering this change. Compared with the results calculated by the material balance method, the results calculated by the flow material balance method are smaller. ② The production of 660 gas wells in the study area during stable production period is verified. Compared with the open flow method, the dynamic reserve allocation method is better, with an error of 0.06%. ③ The new method in this paper is used to allocate production for different types of gas wells. The cumulative production of different types of gas wells shows different degrees of increase. The I, II, III and IV types of gas wells increase by 32.26%, 30.29%, 23.58% and 25.07% respectively. This study provides technical support for dynamic reserve calculation and reasonable production allocation of gas wells in the study area, and has important guiding significance for the formulation of reasonable development plan and economic and efficient development of tight sandstone gas reservoirs.

## Introduction

Reasonable working system of gas well is an important factor affecting economic limit production and ultimate recovery of gas field. However, the production allocation method of conventional gas reservoirs is not applicable because tight sandstone gas reservoir has poor physical properties, small porosity, low permeability, strong heterogeneity and complex seepage mechanism^1^. The open flow method is also the most commonly used method at present. It generally reflects the seepage characteristics of the formation near the bottom of the well in the early stage of production^2^. The gas well production is generally 1/5–1/3 of the open flow^3, 4^. However, the field production shows that the open flow rate does not reflect the matrix productivity of the far-well zone, and it is very unreliable as a production allocation method for unconventional tight sandstone gas reservoirs.

The most commonly used method for calculating the dynamic reserves of gas wells is the material balance method (MBM)^5, 6^. However, it can be invalid when there is no bottom hole pressure data. In order to solve this problem, Mattar analyzed the flow law of gas wells based on the perspective of seepage mechanics, and proposed the flow material balance method (FMB)^7^. It proposed that the decline of bottom hole pressure and formation pressure was equal for closed gas reservoirs in the same time period when the seepage entered the quasi-steady stage. Therefore, bottom hole flowing pressure and wellhead casing pressure can be used to replace formation pressure in the process of calculating dynamic reserves by MBM.

First, Based on the material balance method, considering the changes of viscosity and compressibility with pressure, a modified FMB is established and calculation steps are given in this study. At the same time, the dynamic reserves allocation method of gas wells was established, and it was verified in combination with the production of 660 gas wells during the stable production period from the perspective of gas well productivity. Second, the relationship between dynamic reserves and gas well production is described and establish a simple “reserves-production” allocation model. Finally, the gas wells in the study area were classified, and the productivity of different types of wells was reasonably predicted.

## Geology

**Figure 1:**
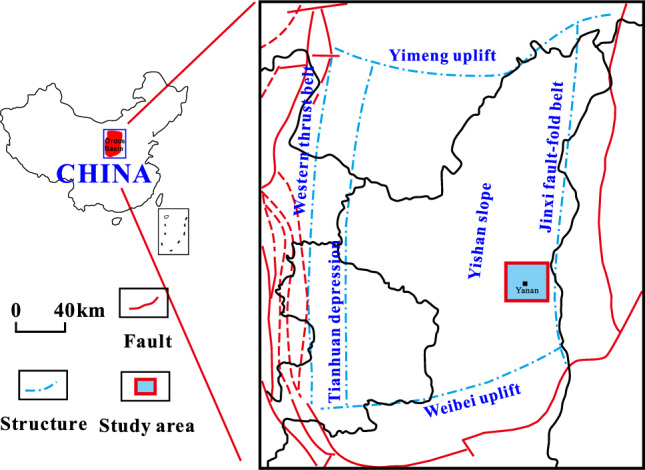
Location of Yan’an Gas Field in Ordos Basin. Created using CorelDRAW-X7 17.1.0.572(https://www.coreldraw.com/cn/).

Ordos basin is a large sedimentary basin with multi-cycle evolution and multi-sedimentary types, and it is about 25 × 10^4 ^km^2^^8^. The internal structure of the basin is relatively simple, without secondary structure, and the tertiary structure is dominated by nasal uplift^9^. As shown in Fig. 1, the study area is located in the southeast of the Yishan slope in the Ordos Basin, where multi-layered rocks are developed, and regional capping layers are widely distributed, which is favorable for the formation and enrichment of gas reservoirs.

Based on the data, 660 wells can be divided into four types according to the classification standard of open flow (Table 1): type I (> 10.0 × 10^4^ m^3^/d/d), type II (4.0–10.0 × 10^4^ m^3^/d), Type III (2.0–4.0 × 10^4^ m^3^/d) and Type IV (with an open flow rate of less than 2.0 × 10^4^ m^3^/d).

**Table 1:** Classification results of gas wells in study area.

Classification (m^3^)	Type	Average/10^4^ m^3^/d	Well number	Content/%
> 10 × 10^4^	I	26.58	103	15.61
4–10 × 10^4^	II	7.3	170	25.76
2–4 × 10^4^	III	2.35	278	42.12
< 2 × 10^4^	IV	1.21	109	16.52

## Methods

At present, the methods for calculating the dynamic reserves of gas wells mainly include material balance method (MBM), production accumulation method (PAM) and elastic two-phase method (ETM)^10^. Since the MBM requires less data in the calculation process and the process is simple, the utilization rate is high^11, 12^.

For a circular closed radial flow, the gas reservoir in the quasi-steady state stage^13, 14^:

1 $$\frac{{\partial \left( {{{\overline{P} } \mathord{\left/ {\vphantom {{\overline{P} } {\overline{{u_{g} }} \overline{{C_{g} }} \overline{Z} }}} \right. \kern-0pt} {\overline{{u_{g} }} \overline{{C_{g} }} \overline{Z} }}} \right)}}{{\partial G_{P} }} = \frac{{\partial \left( {{{\overline{{P_{wf} }} } \mathord{\left/ {\vphantom {{\overline{{P_{wf} }} } {u_{gwf} c_{gwf} \overline{Z}_{wf} }}} \right. \kern-0pt} {u_{gwf} c_{gwf} \overline{Z}_{wf} }}} \right)}}{{\partial G_{P} }}$$

In the FMB established by Mattar, it is assumed that the pressure has no effect on the properties (viscosity and compressibility) of natural gas^7, 15^:

2 $$\partial \left( {\overline{{u_{g} }} \overline{{c_{g} }} } \right) = \partial \left( {u_{gwf} c_{gwf} } \right)$$

3 $$\frac{{\partial \left( {{{\overline{P}} \mathord{\left/ {\vphantom {{\overline{P}} {\overline{Z}}}} \right. \kern-0pt} {\overline{Z}}}} \right)}}{{\partial G_{P} }} = \frac{{\partial \left( {{{\overline{P}_{wf} } \mathord{\left/ {\vphantom {{\overline{P}_{wf} } {\overline{Z}_{wf} }}} \right. \kern-0pt} {\overline{Z}_{wf} }}} \right)}}{{\partial G_{P} }}$$

**Figure 2:**
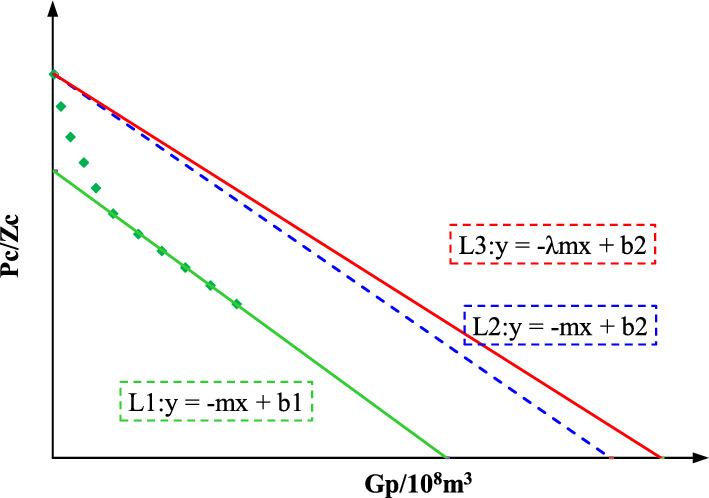
Determination of Dynamic Reserves by Modified FMB Method.

When the reservoir reaches a quasi-steady state, according to the *P*_*wf*_/*Z*_*wf*_ and *G*_*n*_ obtained in production, the data showing a linear trend are fitted, and then draw a parallel line through the *P*_*i*_/*Z*_*i*_ point^16, 17^. The intercept of the parallel line on the *G*_*n*_ coordinate is the dynamic reserves *G*_*i*_ (Fig. 2).

**Figure 3:**
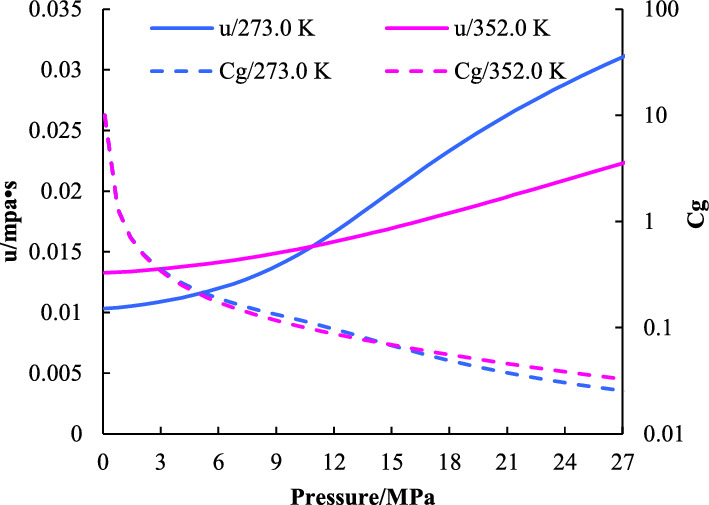
Relationship between pressure and properties.

Based on the natural gas composition in the study area (Table 2), the variation of natural gas properties with pressure is obtained by mathematical simulation method. The results show that the viscosity of natural gas increases with the pressure (Fig. 3), the compressibility decreases with the pressure, and the product of the two decreases with the pressure.

**Table 2:** Natural gas component analysis data of 22 samples.

Number	CH_4_/%	C_2_H_6_/%	C_3_H_8_/%	N_2_/%	CO_2_/%	kg/m^3^	ρ_rel_
N-1	95.37	0.571	0.038	1.048	2.969	0.7119	35.8
N-2	95.302	0.465	0.024	1.484	2.72	0.7104	35.7
N-3	95.299	0.38	0.032	1.965	2.304	0.7074	35.7
N-4	96.396	0.633	0.059	1.721	1.139	0.6964	36.2
N-5	96.469	0.521	0.086	2.436	0.223	0.6858	36.3
N-6	95.865	0.42	0.021	1.368	2.246	0.7055	36.0
N-7	96.211	0.423	0.023	1.379	1.962	0.7007	36.0
N-8	96.358	0.532	0.038	1.451	1.598	0.6977	36.2
N-9	96.706	0.535	0.04	1.024	1.691	0.6967	36.3
N-10	95.603	0.433	0.028	1.432	2.499	0.7074	35.8
N-11	95.769	0.428	0.023	1.588	2.188	0.7045	35.8
N-12	95.792	0.453	0.028	1.508	2.214	0.7046	35.9
N-13	95.730	0.453	0.028	1.514	2.27	0.7053	35.9
N-14	96.396	0.633	0.059	1.721	1.139	0.6946	36.3
N-15	96.082	0.353	0.019	1.319	2.225	0.703	35.9
N-16	95.853	0.43	0.022	1.476	1.992	0.7048	36.1
N-17	96.600	0.775	0.072	1.588	0.900	0.6921	36.5
N-18	94.838	0.564	0.038	0.962	3.594	0.7187	35.6
N-19	95.861	0.59	0.048	1.762	1.698	0.7008	36.0
N-20	95.834	0.479	0.025	1.063	2.586	0.7067	35.9
N-21	95.806	0.437	0.028	1.481	2.244	0.7047	35.9
N-22	96.198	0.42	0.023	1.412	1.945	0.7007	36.0
Min	94.838	0.353	0.019	0.962	0.223	0.6858	35.6
Max	96.706	0.775	0.086	2.436	3.594	0.7187	36.5
Average	95.912	0.502	0.038	1.504	2.007	0.703	35.996

Based on the above derivation, it is proved that the compressibility and viscosity of natural gas change with pressure, then there is an error in the calculation of dynamic reserves of gas wells by the FMB.

4 $$\left| {\partial \left( {\overline{{u_{g} }} \overline{{c_{g} }} } \right)} \right| < \left| {\partial \left( {u_{gwf} c_{gwf} } \right)} \right|$$

Therefore, the slope of the *P*_*wf*_/*Z*_*w*_*—G*_*p*_ is greater than that of the $\overline{P} /\overline{Z} - G_{p}$, and when the formation pressure is small, the difference between the them is proportional to the production pressure difference. Therefore, it is necessary to revise the FMB in order to reduce the error of the dynamic reserves of gas wells.

5 $$\frac{{\partial \left( {{{\overline{P} } \mathord{\left/ {\vphantom {{\overline{P} } {\overline{Z} }}} \right. \kern-0pt} {\overline{Z} }}} \right)}}{{\partial G_{P} }} = \frac{{\partial \left( {\overline{{u_{g} }} \overline{{C_{g} }} } \right)}}{{\partial \left( {u_{gwf} c_{gwf} } \right)}} \cdot \frac{{\partial \left( {{{P_{wf} } \mathord{\left/ {\vphantom {{P_{wf} } {Z_{wf} }}} \right. \kern-0pt} {Z_{wf} }}} \right)}}{{\partial G_{P} }}$$

It is assumed that *P*_*wf-pss*_ and $\overline{{P_{pss} }}$ represent bottom hole pressure and average formation pressure at the beginning of the pseudo-steady state, respectively. In the quasi-steady state, *P*_*wf-pss*_ and $\overline{{P_{pss} }}$ decrease at the same speed, and it can be considered that λ remains unchanged. When the gas well produces, it will reach a quasi-steady state, and the difference between *P*_*i*_ and $\overline{{P_{pss} }}$ is small:

6 $$\frac{{\partial \left( {\overline{{u_{g} }} \overline{{C_{g} }} } \right)}}{{\partial \left( {u_{gwf} c_{gwf} } \right)}} \approx \frac{{\left. {\left( {u_{g} C_{g} } \right)} \right|_{{\overline{{P_{pss} }} }} }}{{\left. {\left( {u_{g} C_{g} } \right)} \right|_{{P_{wf} - pss}} }} \approx \frac{{\left. {\left( {u_{g} C_{g} } \right)} \right|_{{p_{i} }} }}{{\left. {\left( {u_{g} C_{g} } \right)} \right|_{{P_{wf} - pss}} }} = \lambda$$

## Result

### Dynamic reserve allocation method

1. Conventional method

**Figure 4:**
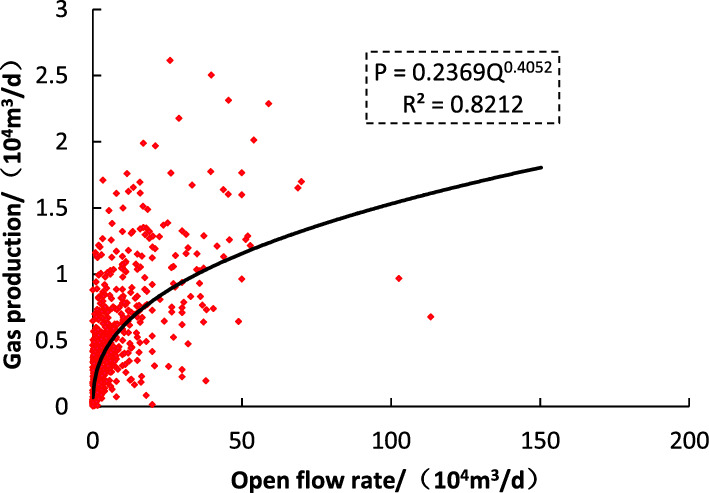
Relationship between open flow and production during stable period.

**Figure 5:**
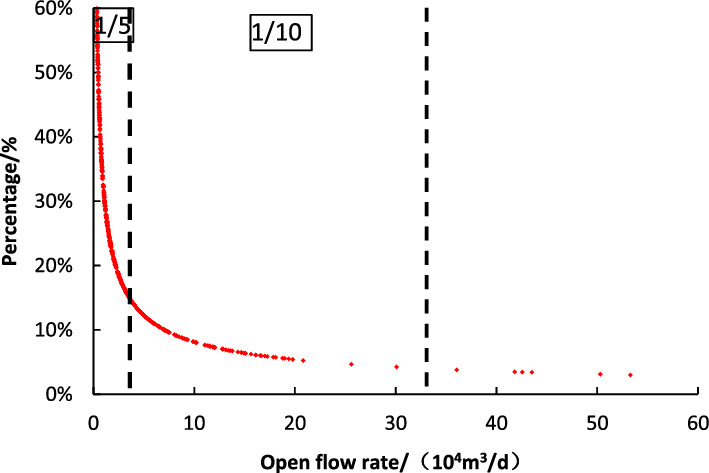
Relationship between distribution coefficient and open flow.

Tight sandstone reservoir with small porosity and low permeability needs stimulated reservoir volume to get industrial exploration^18^. The volume fracturing will form a complex fracture network near the wellbore, thus forming a dual pore medium, and the seepage law will change. According to the relationship between open flow rate and daily production in stable production period of 660 wells in the study area (Fig. 4), the results show that this ratio gradually decreases with the open flow rate (Fig. 5).

(2) Dynamic reserves

Due to the simple operation of the open flow method, it is often used as a production allocation method in the field^19^. However, the open flow obtained in the early stage of production only represents the seepage law of fluid in the fracture zone or high-permeability area near the wellbore. For tight sandstone reservoirs, this allocation method has limitations, often resulting in high production allocation and rapid decline in the field.

**Figure 6:**
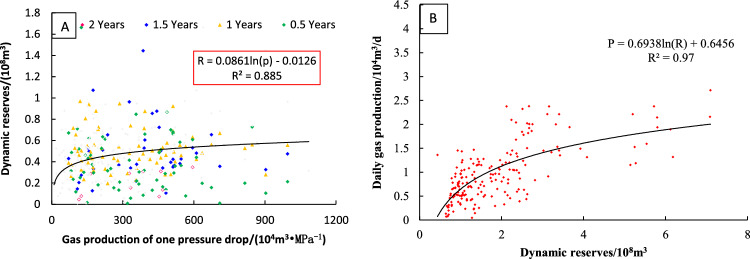
Process for determining a new method of daily gas production (**A**: The relationship between dynamic reserves and well pressure drop; **B**: The relationship between dynamic reserves and Daily gas production).

The dynamic reserves of a single well are one of the important factors that reflect the stable productivity of gas wells. In this study, the mathematical relationship between gas production allocation and open flow is fitted to establish the calculation method of dynamic reserves of tight sandstone gas reservoirs, and a set of fast and operable ‘one curve, two chart’ gas well reasonable production allocation chart is formed. As shown in Fig. 6: the conventional method is used to allocate production to the gas well in the early stage of gas well production (Fig. 4); after a period of gas well production, the dynamic control reserves of single well (Fig. 6A) are obtained. Finally, the (Fig. 6B) chart is used for reasonable production allocation of gas wells.

### Validation

**Figure 7:**
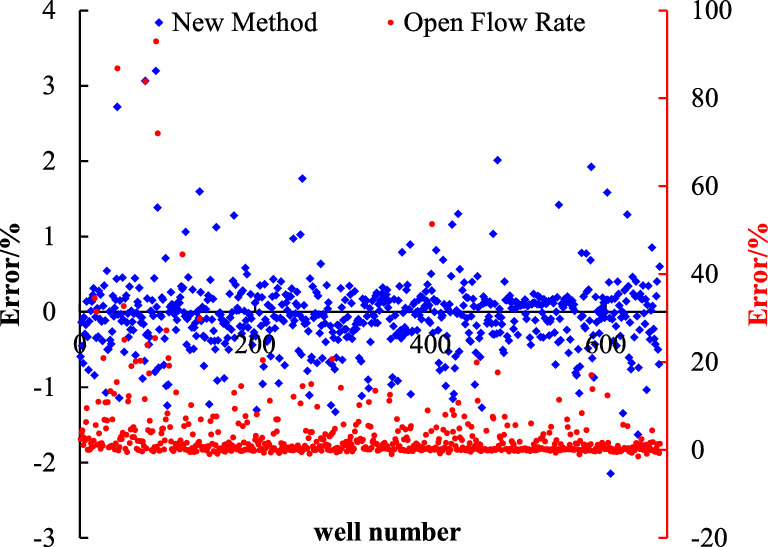
Comparison of conventional allocation and new method.

According to the established dynamic reserve allocation method, the wells in the study area are allocated. Firstly, the dynamic reserves of 660 wells are analyzed and allocated, and the results are compared with the open flow allocation method (Table 3) (More information on the region is provided in the attachment). The error between the calculated results and the daily production during stable production period is analyzed, as shown in Fig. 7.

**Table 3:** Result of conventional allocation and new method.

Number	well	Open flow rate (10^4^ m^3^/d)	Production at stable period (10^4^ m^3^/d)	Conventional allocation (10^4^ m^3^/d)	New method (10^4^ m^3^/d)	Error of conventional allocation (%)	Error of new method (%)
1	S5	4.69	1.45	5.09	2.05	2.51	− 0.60
2	S6	8.94	1.33	6.46	5.54	3.87	− 0.14
3	S207	11.15	2.00	10.92	5.95	4.45	− 0.46
4	Y217	4.24	1.68	3.61	1.13	1.15	− 0.69
5	YQ2	6.10	1.04	3.91	3.62	2.75	− 0.07
6	S229	5.36	1.27	4.09	2.88	2.21	− 0.30
7	Y250	11.96	1.49	10.96	9.03	6.36	− 0.18
8	S12	14.21	1.15	12.20	13.82	9.57	0.13
9	S14	2.01	1.16	1.25	0.83	0.08	− 0.34
10	Y252	6.40	1.91	6.88	1.56	2.61	− 0.77
11	S217	1.50	0.76	0.82	0.72	0.08	− 0.13
12	S226	1.10	0.27	0.35	0.41	0.28	0.15
13	S42	4.37	0.81	2.03	2.05	1.52	0.01
14	Y185	1.96	0.45	0.77	0.87	0.72	0.13
15	Y154	2.00	0.35	0.64	0.78	0.82	0.22
16	Y176	3.90	0.44	1.37	1.80	2.14	0.31
17	S224	51.32	3.61	128.29	19.85	34.55	− 0.85
18	Y169	8.49	1.15	7.62	7.08	5.63	− 0.07
19	Y170	33.35	2.24	72.63	47.38	31.42	− 0.35
20	S205	3.91	0.71	1.54	1.61	1.18	0.04
21	S214	17.59	1.89	22.70	15.24	11.01	− 0.33
22	S221	8.58	0.59	2.80	3.57	3.76	0.28
23	S1	3.90	0.89	2.17	1.96	1.45	− 0.10
24	S11	1.33	0.27	0.35	0.42	0.28	0.23
25	S13	4.50	0.99	2.55	2.21	1.58	− 0.13
26	S15	3.72	1.20	2.39	1.46	0.98	− 0.39
27	S16	41.76	1.94	42.57	45.75	20.94	0.07
28	S18	13.20	0.87	6.86	9.22	6.91	0.34
29	S19	27.73	1.92	22.77	20.10	10.84	− 0.12
30	S20	11.16	2.38	13.96	− 1.01	4.88	− 1.07

Taking the production during the stable production period of gas wells as the criterion, the results calculated by the open flow method are generally larger, resulting in higher gas production allocation and faster decline rate. However, the results determined by the new method are closer to the gas production during the stable production period, with reasonable production allocation, smaller gas well decreasing rate and higher recovery rate. Compared with the results of the open flow method with the average error of 1.15%, the calculation error of the plate method with the average of 0.06% is smaller.

## Discussion

The wells in the study area were divided into four types based on the mercury injection parameters, curve shape and production data^8, 20^.

### Type-I

**Figure 8:**
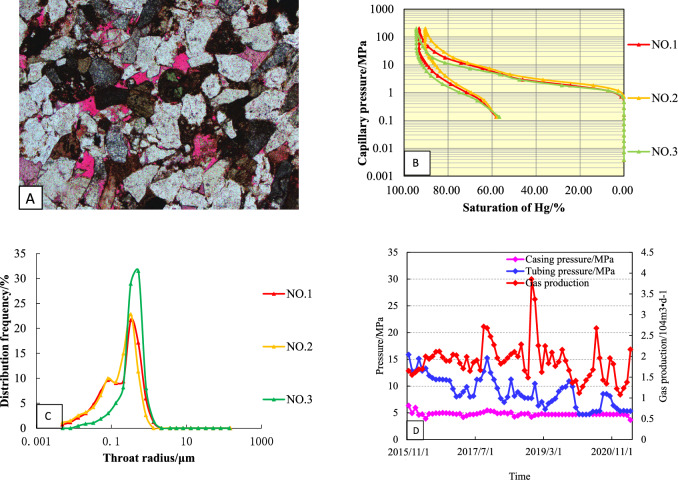
Reservoir characteristics of Type-I well (**A**: Pore throat structures; **B**: Mercury injection curve; **C**: Pore—throat patterns; **D**: Production Curve).

The curve is characterized by high saturation of mercury, capillary pressure curve to the left, high on the left and low on the right, and a platform curve, with an average porosity of 6.6% and an average permeability of 0.26 × 10^−3^ μm^2^ (Fig. 8A). The displacement pressure of this kind of reservoir is small, ranging from 0.29 to 1.16 MPa, with an average of 0.59 MPa (Fig. 8B). The maximum mercury saturation is between 90.24 and 94.35%, with an average of 92.34%. The pore throat radius is mainly distributed in 0.062–0.38 μm, with an average of 0.225 μm (Fig. 8C). The pore throat of this kind of pore structure is coarse, which is the best type of reservoir structure in the study area.

Type I wells in the study area have the highest initial production, slow pressure drop, long stable production time, and good production stability under low pressure conditions (Fig. 8D). Well S1 is a typical type I well with an open flow rate of 38.53 × 10^4^ m^3^/month. It has been in production since January 2015. From the production curve, it can be seen that the average monthly production of gas wells is 23.15 × 10^4^ m^3^/month, the water production is at a low level, the average monthly production is 0.14 m^3^/month, and the water–gas ratio is maintained at 0.01 (m^3^/10^4^ m^3^) until to March 2019. In the second stage (March 2019–December 2019), the casing pressure, the oil pressure and the monthly gas production decreased rapidly. In the third stage (December 2019–April 2022), the monthly gas was maintained at a low level, and the monthly water production was higher, with casing pressure maintained at about 7.8 MPa and oil pressure maintained at about 5.6 MPa.

**Figure 9:**
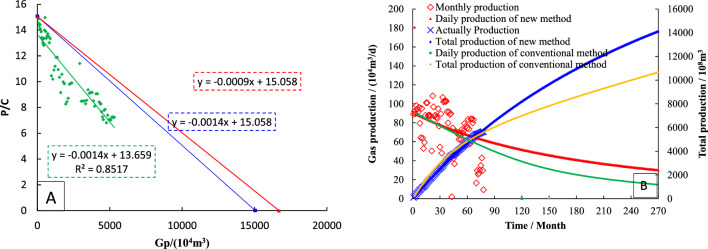
Dynamical properties of type-I wells (**A**: Dynamic reserves; **B**: Production prediction).

The *P*_*c*_/*Z*_*c*_ ~ *G*_*p*_ curve is drawn by production data, and the data points showing a straight line trend are linearly fitted. The slope of the straight line is − 0.0014, and it is used as a straight line through the *P*_*i*_/*Z*_*i*_ point. The intercept in the horizontal coordinate is 1.51 × 10^8^ m^3^, which is the dynamic reserve of S1 well determined by the FMB (Fig. 9A).

Based on the − λ (− λm = − 0.9), it can be gotten the intercept of straight line through the *P*_*i*_/*Z*_*i*_ point is 1.67 × 10^8^ m^3^, which is the dynamic reserves of well S1 determined by modified FMB.

Using dynamic reserves to rationally allocate gas well production (Fig. 9B), the historical matching results of type I well production and cumulative production are well^21^. The abandoned production was set to 1000 m^3^/d, and the decline prediction equation was used to simulate the production. As of April 2037, the cumulative production was 1400^22^ 2.56 × 10^4^ m^3^, and the monthly decline rate was 0.016%.

### Type-II

**Figure 10:**
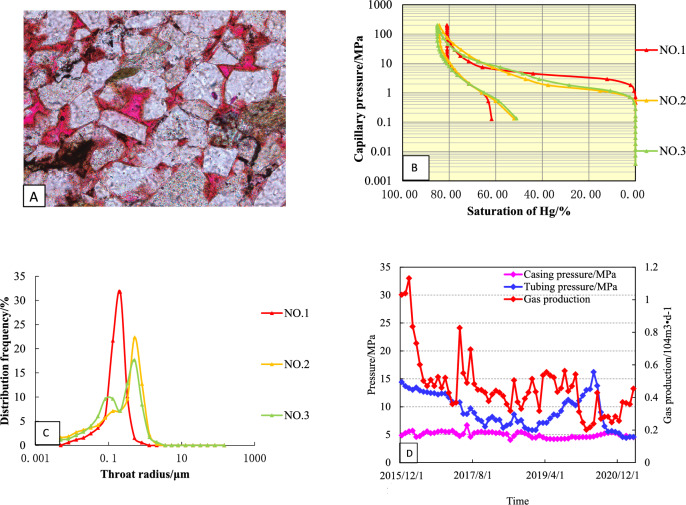
Reservoir characteristics of Type-II well (**A**: Pore throat structures; **B**: Mercury injection curve; **C**: Pore—throat patterns; **D**: Production Curve).

The mercury injection curve of this type of reservoir is characterized by: high—higher mercury saturation, slightly concave to the left, gently sloping, and the platform is shorter (Fig. 10A). The porosity is 3.5–8.97%, and the permeability is 0.05–0.21 × 10^−3^ μm^2^. Compared with the type I reservoir, the displacement pressure is higher, mainly distributed in 0.28–1.82 MPa, with an average of 0.7 MPa; The maximum mercury saturation ranges from 80.9 to 87.78%, with an average of 84.37% (Fig. 10B); the throat distribution is skewed by coarse crookedness, which is a better type II reservoir in the study area (Fig. 10C).

S2 is a typical type II well. The open flow rate of the well test gas is 23.78 × 10^4^ m^3^/d, the original formation pressure is 19.84 MPa, and the production is allocated according to 30 × 10^4^ m^3^/d at the beginning of the production test (Fig. 10D). Due to the large pressure fluctuation in the test production, the gas production is difficult to stabilize. After adjusting the working system, the gas production is gradually reduced to about 16.52 × 10^4^ m^3^/month, and the water production is 0.1–0.3 m^3^/month. When the gas production was reduced to 13.25 × 10^4^ m^3^/month, the tubing pressure decreased from 16.3 to 5.8 MPa, while the casing pressure was basically stable. As of April 2022, the cumulative gas production was 2985.26 × 10^4^ m^3^.

**Figure 11:**
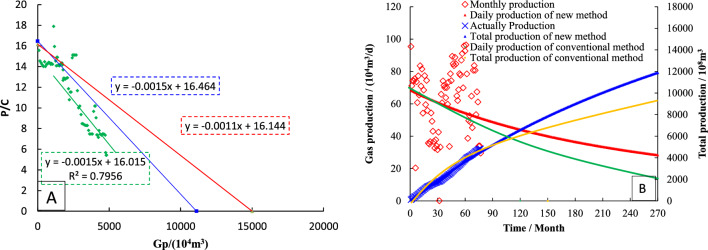
Dynamical properties of type-II wells (**A**: Dynamic reserves; **B**: Production prediction).

The *P*_*c*_/*Z*_*c*_ ~ *G*_*p*_ curve is drawn by production data, and the data points showing a straight line trend are linearly fitted. The slope of the straight line is − 0.015, and it is used as a straight line through the *P*_*i*_/*Z*_*i*_. The intercept in the horizontal coordinate is 1.11 × 10^8^ m^3^, which is the dynamic reserve of S2 determined by the FMB (Fig. 11A).

Based on the − λ (− λm = − 0.67), it can be gotten the intercept of straight line through the *P*_*i*_/*Z*_*i*_ point is 1.51 × 10^8^ m^3^, which is the dynamic reserves of well S2 determined by modified FMB.

The dynamic reserves is used to rationally allocate gas well production, the historical matching results of type II production and cumulative production are good (Fig. 11B). The abandoned production (1000 m^3^/d) was set, and the decline prediction equation was used to simulate the future production of gas wells. As of April 2037, the cumulative production was 12,056.78 × 10^4^ m^3^, and the monthly decline rate was 0.03%.

### Type III

**Figure 12:**
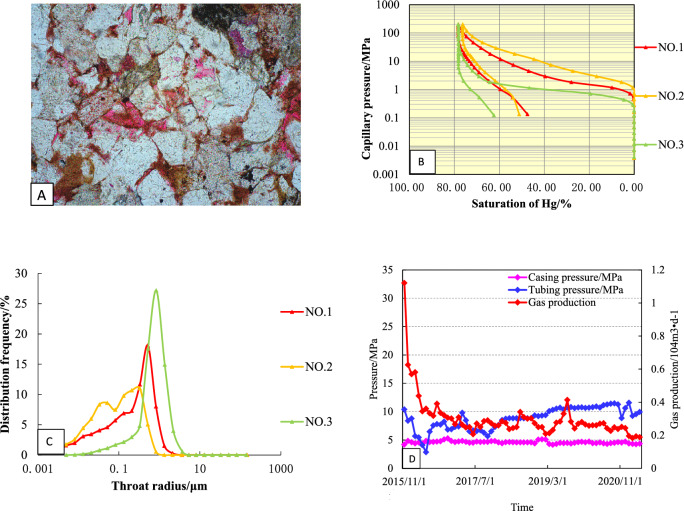
Reservoir characteristics of Type-III well (**A**: Pore throat structures; **B**: Mercury injection curve; **C**: Pore—throat patterns; **D**: Production Curve).

The porosity of this kind of sandstone reservoir ranges from 3 to 8.7%, the permeability ranges from 0.02 to 0.636 × 10^−3^ μm^2^, and the displacement pressure is medium, ranging from 0.43 to 1.82 MPa, with an average of 0.87 MPa (Fig. 12A). The platform of the capillary pressure curve is not obvious, showing a steep slope, the throat sorting is poor, and the distribution of the pore throat radius is from 0.02 to 0.58 μm, which is on the side of fine skewness, and it is the poorer type III reservoir in the study area (Fig. 12B,C).

S3 is a typical class III well in this area, with an open flow rate of 16.57 × 10^4^ m^3^/d. It has been in production since January 2015 (Fig. 12D). It can be seen from the production curve that the average monthly production of gas wells was 35.6 × 10^4^ m^3^/month in the early stage of production (December 2014–March 2015), the water production was at a low level, and the gas production decreased rapidly until to 13.24 m^3^/month. During the second stage of production, the casing pressure decreased rapidly and the monthly gas production remained unchanged. In the third stage of production (February 2017–April 2021), the average production was 7.5 m^3^/month. Up to now, the cumulative gas production of S3 is 2062.51 × 10^4^ m^3^.

**Figure 13:**
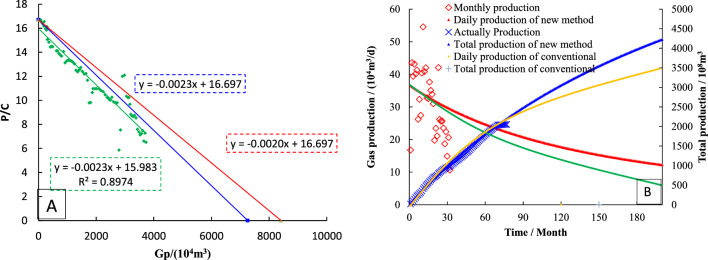
Dynamical properties of type-III wells (**A**: Dynamic reserves; **B**: Production prediction).

The *P*_*c*_/*Z*_*c*_ ~ *G*_*p*_ curve is drawn by production data, and the data points showing a straight line trend are linearly fitted. The slope of the straight line is − 0.0023, and it is used as a straight line through the *P*_*i*_/*Z*_*i*_. The intercept in the horizontal coordinate is 0.76 × 10^8^ m^3^, which is the dynamic reserve of S3 determined by the FMB (Fig. 13A).

Based on the − λ (− λm = − 0.92), it can be gotten the intercept of straight line through the *P*_*i*_/*Z*_*i*_ point is 0.84 × 10^8^ m^3^, which is the dynamic reserves of well S3 determined by modified FMB.

The dynamic reserves is used to rationally allocate gas wells (Fig. 13B), the historical matching results of type III well production and cumulative production are good. The abandoned production (1000 m^3^/d) was set, and the decline prediction equation was used to simulate the future production. As of February 2028, the cumulative production was 4325.69 × 10^4^ m^3^, and the monthly decline rate was 0.08%.

### Type IV

**Figure 14:**
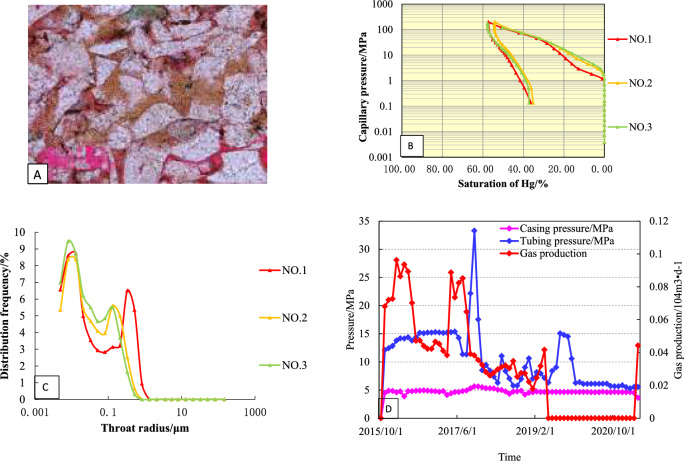
Reservoir characteristics of Type-IV well (**A**: Pore throat structures; **B**: Mercury injection curve; **C**: Pore—throat patterns; **D**: Production Curve).

The porosity of this kind of reservoir is 0.79–4.53%, and the permeability is 0.001–0.167 × 10^−3^ μm^2^ (Fig. 14A). The capillary pressure curve is narrow, showing a steep slope distributed in the upper half of the figure, and there is no obvious wide platform section (Fig. 14B). The average displacement pressure is 2.76 MPa, and the distribution of the throat is fine crookedness with poor sorting (Fig. 14C).

S4 is a typical class IV well in this area, with an open flow rate of 9.3 × 10^4^ m^3^/d. It has been in production since October 2015 (Fig. 14D). The production of gas wells was 30 × 10^4^ m^3^/month in the early stage of production (October 2015–December 2015). During the second stage of production (July 2017–March 2019), the tubing pressure and the gas production decreased rapidly. In the third stage of production (April 2019–April 2022), the gas production maintain a low level. Up to now, the cumulative gas production of S3 is 986.32 × 10^4^ m^3^.

**Figure 15:**
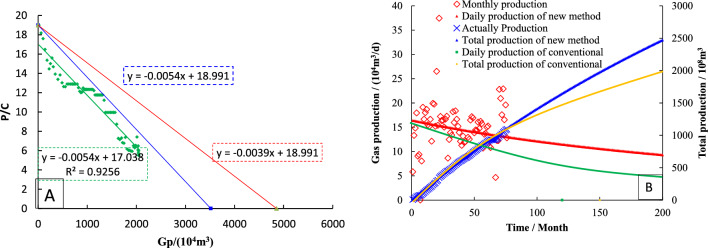
Dynamical properties of type-IV wells (**A**: Dynamic reserves; **B**: Production prediction).

The Pc/Zc ~ Gp curve (Fig. 15A) is drawn by production data, and the data points showing a straight line trend are linearly fitted. The slope of the straight line is -0.0054, and it is used as a straight line through the Pi/Zi. The intercept in the horizontal coordinate is 0.35 × 10^8^ m^3^, which is the dynamic reserve of S4 determined by the FMB.

Based on the − λ (− λm = − 0.69), it can be gotten the intercept of straight line through the *P*_*i*_/*Z*_*i*_ point is 0.49 × 10^8^ m^3^, which is the dynamic reserves of well S4 determined by modified FMB.

The dynamic reserves is used to rationally allocate gas wells (Fig. 15B), the historical matching results of type III well production and cumulative production are good. The abandoned production (1000 m^3^/d) was set, and the decline prediction equation was used to simulate the future production. As of February 2027, the cumulative production was 2501.29 × 10^4^ m^3^, and the monthly decline rate was 0.13%.

The new method in this paper is used to allocate production for different types of gas wells. The results are shown in the Table 4. The cumulative production of different types of gas wells shows different degrees of increase. The I, II, III and IV types of gas wells increase by 32.26%, 30.29%, 23.58% and 25.07% respectively. Among them, the single well production of the II and III types of gas wells increased by 2 times. The average production of the four types of gas wells is increased by 27.80%, which optimized the decreasing rate of gas wells, increased the cumulative production and achieved the purpose of improving the recovery rate.

**Table 4:** The results of optimized production allocation of different types of wells.

Well type	Daily production of conventional method/10^4^ m^3^/d	Total production of conventional method/10^8^ m^3^	Daily production of new method/10^4^ m^3^/d	Total production of new method/10^8^ m^3^	Improvement of daily production/%	Improvement of total production/%
Type-I	1653.23	10,587.12	2158.51	14,002.56	30.56	32.26
Type-II	2002.19	9253.48	4019.54	12,056.78	100.76	30.29
Type-III	501.56	3500.23	1006.79	4325.69	100.73	23.58
Type-IV	436.59	1999.85	780.51	2501.29	78.77	25.07
Average	1148.39	6335.17	1991.34	8221.58	77.71	27.80

## Conclusion

1. The theoretical calculation and experiment results show that the viscosity of natural gas increases rapidly with the pressure, the compressibility decreases rapidly with the pressure. The product of the two decreases with pressure. Considering the changes of viscosity and compressibility, a modified FMB is established and calculation steps are given.
2. The dynamic reserves allocation method of gas wells was established, and it was verified in combination with the production of 660 gas wells in the study area during the stable production period. Compared with conventional production allocation, the results of dynamic reserve allocation method are closer to the production in the stable production period, with an average error of 0.06%.
3. The wells in the study area were divided into four types based on the mercury injection parameters, curve shape and production data. The new method in this paper is used to allocate production for different types of gas wells. The cumulative production of different types of gas wells shows different degrees of increase. The I, II, III and IV types of gas wells increase by 32.26%, 30.29%, 23.58% and 25.07% respectively.

### Supplementary Information

Supplementary Information.

## Data Availability

The datasets used and analysed during the current study available from the corresponding author on reasonable request.

## List of symbols

$\overline{P}$: Average reservoir pressure, MPa
$\overline{{u_{g} }}$: Viscosity of natural gas under average formation pressure, mPa s
$\overline{{C_{g} }}$: Natural gas compressibility under average formation pressure, MPa^−^^1^
$\overline{Z}$: Natural gas deviation coefficient under average formation pressure
$G_{p}$: Accumulated gas production, 10^4^ m^3^
$u_{gwf}$: Viscosity of natural gas under bottom hole pressure, mPa s
$C_{gwf}$: Compression coefficient of natural gas under bottom hole pressure, MPa^−^^1^
