# Supplementary material for: Study on development methods of different types of gas wells in tight sandstone gas reservoirs
Source: Sci Rep. 2023 Sep 29;13:16380. doi: 10.1038/s41598-023-43640-7 (PMC10541403; doi:10.1038/s41598-023-43640-7)
Supplement: Supplementary file 1 — Supplementary Information. [file 41598_2023_43640_MOESM1_ESM.docx]

**Study on development methods of different types of gas wells in tight sandstone gas reservoirs**

Jie He, Fangxin Song^2^, Dong Hao^3^, Mingming Liu^2^, Zhiwei Liu^3^, Heng Zhang^3^, Shenghong Xie^3^, Xiqiang Wang^3^, Yushuang Zhu ^1^

1. State Key Laboratory of Continental Dynamics/Department of Geology, Northwest University, Xi’an, 710069, China;

2. No.10 Production Plant, PetroChina Changqing Oilfield Company, Qingyang 745100, China.

3. No.7 Production Plant, PetroChina Changqing Oilfield Company, Xifeng 745000, China.

**Corresponding author:** Yushuang Zhu, Professor, Doctoral Supervisor, Northwest University, China. **E-mail address:** [petroleum_gas@163.com](mailto:petroleum_gas@163.com).

The dynamic reserves of 660 wells by conventional production allocation and new method are as follow:

| Number | well | Open flow rate (10^4^m^3^/d) | Production  (10^4^m^3^/d) | Conventional allocation  (10^4^m^3^/d) | New  method  (10^4^m^3^/d) | Error of conventional  allocation  (%) | Error of New  method (%) |
| --- | --- | --- | --- | --- | --- | --- | --- |
| 1 | S5 | 4.70 | 1.45 | 5.09 | 2.05 | 2.51 | -0.60 |
| 2 | S6 | 8.94 | 1.33 | 6.46 | 5.54 | 3.87 | -0.14 |
| 3 | S207 | 11.15 | 2.00 | 10.92 | 5.95 | 4.45 | -0.46 |
| 4 | Y217 | 4.24 | 1.68 | 3.61 | 1.13 | 1.15 | -0.69 |
| 5 | YQ2 | 6.10 | 1.04 | 3.91 | 3.62 | 2.75 | -0.07 |
| 6 | S229 | 5.36 | 1.27 | 4.09 | 2.88 | 2.21 | -0.30 |
| 7 | Y250 | 11.96 | 1.49 | 10.96 | 9.03 | 6.36 | -0.18 |
| 8 | S12 | 14.21 | 1.15 | 12.20 | 13.82 | 9.57 | 0.13 |
| 9 | S14 | 2.01 | 1.16 | 1.25 | 0.83 | 0.08 | -0.34 |
| 10 | Y252 | 6.40 | 1.91 | 6.88 | 1.56 | 2.61 | -0.77 |
| 11 | S217 | 1.50 | 0.76 | 0.82 | 0.72 | 0.08 | -0.13 |
| 12 | S226 | 1.10 | 0.27 | 0.35 | 0.41 | 0.28 | 0.15 |
| 13 | S42 | 4.37 | 0.81 | 2.03 | 2.05 | 1.52 | 0.01 |
| 14 | Y185 | 1.96 | 0.45 | 0.77 | 0.87 | 0.72 | 0.13 |
| 15 | Y154 | 2.00 | 0.35 | 0.64 | 0.78 | 0.82 | 0.22 |
| 16 | Y176 | 3.90 | 0.44 | 1.37 | 1.80 | 2.14 | 0.31 |
| 17 | S224 | 51.32 | 3.61 | 128.29 | 19.85 | 34.55 | -0.85 |
| 18 | Y169 | 8.49 | 1.15 | 7.62 | 7.08 | 5.63 | -0.07 |
| 19 | Y170 | 33.35 | 2.24 | 72.63 | 47.38 | 31.42 | -0.35 |
| 20 | S205 | 3.91 | 0.71 | 1.54 | 1.61 | 1.18 | 0.04 |
| 21 | S214 | 17.59 | 1.89 | 22.70 | 15.24 | 11.01 | -0.33 |
| 22 | S221 | 8.58 | 0.59 | 2.80 | 3.57 | 3.76 | 0.28 |
| 23 | S1 | 3.90 | 0.89 | 2.17 | 1.96 | 1.45 | -0.10 |
| 24 | S11 | 1.33 | 0.27 | 0.35 | 0.42 | 0.28 | 0.23 |
| 25 | S13 | 4.50 | 0.99 | 2.55 | 2.21 | 1.58 | -0.13 |
| 26 | S15 | 3.72 | 1.20 | 2.39 | 1.46 | 0.98 | -0.39 |
| 27 | S16 | 41.76 | 1.94 | 42.57 | 45.75 | 20.94 | 0.07 |
| 28 | S18 | 13.20 | 0.87 | 6.86 | 9.22 | 6.91 | 0.34 |
| 29 | S19 | 27.73 | 1.92 | 22.77 | 20.10 | 10.84 | -0.12 |
| 30 | S20 | 11.16 | 2.38 | 13.96 | -1.01 | 4.88 | -1.07 |
| 31 | S21 | 11.18 | 0.63 | 4.90 | 7.54 | 6.75 | 0.54 |
| 32 | S23 | 25.21 | 2.38 | 28.39 | 14.17 | 10.93 | -0.50 |
| 33 | S24 | 3.48 | 1.40 | 2.19 | 1.09 | 0.56 | -0.50 |
| 34 | S3 | 2.45 | 0.65 | 1.08 | 1.08 | 0.66 | 0.00 |
| 35 | S36 | 23.72 | 2.04 | 29.43 | 18.35 | 13.42 | -0.38 |
| 36 | S37 | 4.18 | 1.37 | 2.69 | 1.55 | 0.97 | -0.43 |
| 37 | S38 | 17.56 | 1.84 | 14.60 | 10.74 | 6.93 | -0.26 |
| 38 | S39 | 2.64 | 0.39 | 0.72 | 0.87 | 0.85 | 0.21 |
| 39 | S4 | 26.57 | 1.78 | 24.53 | 23.10 | 12.79 | -0.06 |
| 40 | S40 | 1.32 | 0.46 | 0.50 | 0.53 | 0.10 | 0.05 |
| 41 | S41 | 3.64 | 0.59 | 1.48 | 1.71 | 1.50 | 0.16 |
| 42 | S47 | 26.53 | 1.20 | 19.76 | 28.38 | 15.47 | 0.44 |
| 43 | S8 | 150.37 | 0.00 | 0.00 | 0.00 | 86.79 | 2.72 |
| 44 | S9 | 0.97 | 0.51 | 0.45 | 0.43 | -0.12 | -0.04 |
| 45 | Y196 | 4.71 | 2.21 | 3.72 | -0.53 | 0.68 | -1.14 |
| 46 | Y202 | 7.40 | 0.77 | 2.68 | 3.07 | 2.49 | 0.15 |
| 47 | S17 | 8.86 | 1.15 | 4.62 | 4.46 | 3.02 | -0.03 |
| 48 | S204 | 6.16 | 1.10 | 3.80 | 3.31 | 2.45 | -0.13 |
| 49 | S206 | 7.54 | 0.49 | 2.40 | 3.48 | 3.89 | 0.45 |
| 50 | S209 | 68.86 | 2.65 | 89.49 | 79.69 | 32.72 | -0.11 |
| 51 | S210 | 52.85 | 2.99 | 78.04 | 36.35 | 25.09 | -0.53 |
| 52 | S220 | 1.50 | 0.33 | 0.43 | 0.50 | 0.30 | 0.17 |
| 53 | S223 | 7.86 | 0.76 | 3.41 | 4.13 | 3.47 | 0.21 |
| 54 | S225 | 0.52 | 0.52 | 0.28 | 0.24 | -0.47 | -0.15 |
| 55 | S234 | 31.30 | 1.86 | 22.39 | 21.84 | 11.02 | -0.02 |
| 56 | S260 | 27.14 | 2.19 | 29.02 | 19.17 | 12.24 | -0.34 |
| 57 | Y120 | 4.02 | 0.34 | 0.87 | 1.17 | 1.60 | 0.33 |
| 58 | Y161 | 13.15 | 1.66 | 9.44 | 7.21 | 4.69 | -0.24 |
| 59 | Y162 | 34.85 | 1.89 | 37.87 | 38.20 | 19.07 | 0.01 |
| 60 | S48 | 8.17 | 1.25 | 4.96 | 4.30 | 2.96 | -0.13 |
| 61 | S53 | 10.57 | 1.45 | 9.03 | 6.78 | 5.22 | -0.25 |
| 62 | S53-1 | 7.44 | 1.06 | 4.19 | 4.07 | 2.96 | -0.03 |
| 63 | S7 | 12.57 | 0.44 | 2.40 | 3.47 | 4.50 | 0.44 |
| 64 | S207-1 | 45.89 | 2.98 | 63.13 | 26.61 | 20.17 | -0.58 |
| 65 | S215 | 2.08 | 0.63 | 0.80 | 0.79 | 0.28 | -0.01 |
| 66 | S216 | 6.56 | 0.74 | 2.32 | 2.62 | 2.11 | 0.13 |
| 67 | S219-1 | 0.98 | 0.29 | 0.29 | 0.32 | 0.01 | 0.09 |
| 68 | S248 | 4.73 | 0.45 | 1.35 | 1.78 | 2.03 | 0.31 |
| 69 | S248-1 | 37.18 | 2.03 | 43.22 | 40.33 | 20.31 | -0.07 |
| 70 | S248-3 | 5.22 | 1.05 | 3.13 | 2.68 | 1.97 | -0.14 |
| 71 | S261 | 0.28 | 0.42 | 0.28 | 0.26 | -0.32 | -0.08 |
| 72 | S261-1 | 5.79 | 1.82 | 5.40 | 0.61 | 1.97 | -0.89 |
| 73 | S56 | 3.42 | 0.95 | 1.84 | 1.53 | 0.94 | -0.17 |
| 74 | S208 | 26.39 | 2.61 | 32.84 | 11.41 | 11.58 | -0.65 |
| 75 | S211 | 116.50 | 0.00 | 0.00 | 0.00 | 83.83 | 3.06 |
| 76 | S211-1 | 2.43 | 0.96 | 1.34 | 0.93 | 0.40 | -0.31 |
| 77 | S212 | 36.78 | 1.76 | 43.78 | 51.30 | 23.85 | 0.17 |
| 78 | S213 | 10.23 | 1.51 | 8.31 | 5.95 | 4.52 | -0.28 |
| 79 | S231 | 31.37 | 2.32 | 42.70 | 24.13 | 17.39 | -0.43 |
| 80 | S232 | 16.54 | 1.90 | 14.59 | 9.64 | 6.68 | -0.34 |
| 81 | S244 | 8.19 | 1.05 | 5.27 | 5.32 | 4.03 | 0.01 |
| 82 | S244-1 | 1.24 | 0.49 | 0.49 | 0.49 | 0.01 | 0.00 |
| 83 | S249 | 10.67 | 0.95 | 5.06 | 5.83 | 4.32 | 0.15 |
| 84 | S249-1 | 0.12 | 0.30 | 0.20 | 0.18 | -0.34 | -0.06 |
| 85 | S249-2 | 1.63 | 0.01 | 0.02 | 0.02 | 0.63 | 0.44 |
| 86 | S249-3 | 39.59 | 2.80 | 74.14 | 14.84 | 25.45 | -0.80 |
| 87 | Y127 | 128.87 | 0.00 | 0.00 | 0.00 | 92.92 | 3.19 |
| 88 | Y217-1 | 2.59 | 0.59 | 1.00 | 1.06 | 0.71 | 0.06 |
| 89 | S213-1 | 113.36 | 1.50 | 109.69 | 261.36 | 72.01 | 1.38 |
| 90 | S213-2 | 3.43 | 2.09 | 2.09 | 0.54 | 0.00 | -0.74 |
| 91 | S213-3 | 1.31 | 0.80 | 0.64 | 0.49 | -0.20 | -0.24 |
| 92 | S248-2 | 4.24 | 0.86 | 2.14 | 2.05 | 1.48 | -0.04 |
| 93 | S246 | 12.47 | 1.38 | 6.54 | 6.13 | 3.75 | -0.06 |
| 94 | S246-1 | 14.74 | 0.97 | 5.58 | 6.63 | 4.76 | 0.19 |
| 95 | S246-2 | 10.29 | 0.96 | 5.68 | 6.60 | 4.93 | 0.16 |
| 96 | S250 | 2.81 | 1.50 | 1.69 | 0.50 | 0.12 | -0.70 |
| 97 | S250-1 | 8.06 | 1.67 | 5.32 | 3.43 | 2.19 | -0.35 |
| 98 | S250-2 | 14.04 | 0.35 | 2.74 | 4.68 | 6.94 | 0.71 |
| 99 | S262 | 45.61 | 3.19 | 89.80 | 1.44 | 27.17 | -0.98 |
| 100 | S262-1 | 15.94 | 2.66 | 24.89 | -6.17 | 8.36 | -1.25 |
| 101 | S263 | 37.33 | 3.05 | 66.82 | 2.28 | 20.94 | -0.97 |
| 102 | S263-1 | 36.17 | 1.94 | 39.09 | 38.76 | 19.19 | -0.01 |
| 103 | S247 | 10.25 | 1.09 | 6.72 | 7.14 | 5.18 | 0.06 |
| 104 | S247-1 | 4.23 | 0.52 | 1.52 | 1.91 | 1.92 | 0.26 |
| 105 | S2 | 1.58 | 0.41 | 0.50 | 0.55 | 0.24 | 0.10 |
| 106 | S61 | 0.65 | 0.24 | 0.21 | 0.23 | -0.11 | 0.10 |
| 107 | S56-1 | 0.75 | 0.41 | 0.32 | 0.32 | -0.23 | -0.01 |
| 108 | S219-2 | 2.34 | 0.71 | 0.92 | 0.89 | 0.30 | -0.03 |
| 109 | S222 | 3.71 | 0.70 | 1.71 | 1.81 | 1.43 | 0.06 |
| 110 | S25 | 45.43 | 2.47 | 34.85 | 26.49 | 13.09 | -0.24 |
| 111 | S27 | 5.00 | 1.04 | 2.03 | 1.81 | 0.95 | -0.11 |
| 112 | S28 | 4.36 | 0.66 | 1.45 | 1.64 | 1.22 | 0.13 |
| 113 | S57 | 4.89 | 0.84 | 2.16 | 2.20 | 1.58 | 0.02 |
| 114 | S58 | 0.52 | 0.22 | 0.25 | 0.28 | 0.16 | 0.12 |
| 115 | Y186 | 7.32 | 0.75 | 2.37 | 2.83 | 2.17 | 0.19 |
| 116 | Y186-1 | 6.06 | 0.27 | 0.67 | 0.90 | 1.44 | 0.34 |
| 117 | YP1 | 102.71 | 2.61 | 118.73 | 154.85 | 44.52 | 0.30 |
| 118 | S51N | 2.20 | 0.27 | 0.50 | 0.66 | 0.86 | 0.32 |
| 119 | Y154E | 20.00 | 1.53 | 9.88 | 9.82 | 5.48 | -0.01 |
| 120 | Y154E-1 | 1.30 | 0.59 | 0.67 | 0.63 | 0.14 | -0.06 |
| 121 | Y154E-2 | 21.50 | 0.00 | 0.00 | 0.00 | 7.17 | 1.06 |
| 122 | Y154E-3 | 10.00 | 1.94 | 5.21 | 2.94 | 1.68 | -0.43 |
| 123 | Y154E-4 | 0.60 | 0.54 | 0.51 | 0.46 | -0.06 | -0.10 |
| 124 | Y154N | 20.80 | 0.81 | 5.57 | 8.11 | 5.89 | 0.46 |
| 125 | Y154N-2 | 0.10 | 0.24 | 0.23 | 0.22 | -0.03 | -0.04 |
| 126 | Y176W | 0.10 | 0.73 | 0.38 | 0.19 | -0.49 | -0.51 |
| 127 | Y176W-2 | 40.50 | 2.06 | 23.17 | 22.52 | 10.22 | -0.03 |
| 128 | Y252-1 | 3.20 | 1.04 | 1.51 | 1.19 | 0.45 | -0.21 |
| 129 | Y252-2 | 12.30 | 0.81 | 3.67 | 4.74 | 3.54 | 0.29 |
| 130 | Y252-3 | 0.20 | 0.73 | 0.38 | 0.19 | -0.48 | -0.49 |
| 131 | Y252-4 | 1.90 | 0.92 | 1.01 | 0.80 | 0.09 | -0.21 |
| 132 | Y252N | 5.00 | 1.10 | 2.12 | 1.81 | 0.93 | -0.15 |
| 133 | Y252N-2 | 0.20 | 0.85 | 0.58 | 0.39 | -0.32 | -0.32 |
| 134 | Y310 | 5.20 | 0.90 | 1.93 | 1.90 | 1.13 | -0.01 |
| 135 | Y310-1 | 5.55 | 0.89 | 2.29 | 2.32 | 1.57 | 0.01 |
| 136 | Y338 | 5.70 | 1.00 | 2.89 | 2.65 | 1.90 | -0.08 |
| 137 | Y338-2 | 120.00 | 0.00 | 0.00 | 0.00 | 29.88 | 1.60 |
| 138 | Y338-3 | 17.50 | 1.16 | 9.22 | 11.60 | 6.95 | 0.26 |
| 139 | Y338-4 | 3.00 | 0.83 | 1.47 | 1.29 | 0.77 | -0.12 |
| 140 | Y338N | 0.10 | 0.06 | 0.06 | 0.07 | 0.07 | 0.06 |
| 141 | Y338N-1 | 3.00 | 1.12 | 1.55 | 1.05 | 0.38 | -0.32 |
| 142 | Y338N-2 | 5.00 | 1.77 | 2.91 | 0.82 | 0.64 | -0.72 |
| 143 | Y338N-3 | 3.50 | 1.19 | 1.81 | 1.18 | 0.51 | -0.34 |
| 144 | Y338S | 0.30 | 0.23 | 0.24 | 0.25 | 0.04 | 0.03 |
| 145 | Y338S-1 | 0.25 | 0.43 | 0.39 | 0.36 | -0.08 | -0.09 |
| 146 | Y338S-2 | 4.00 | 0.53 | 1.02 | 1.18 | 0.92 | 0.15 |
| 147 | Y339W | 2.90 | 0.34 | 0.58 | 0.70 | 0.72 | 0.21 |
| 148 | Y340 | 1.48 | 1.79 | 0.18 | -0.04 | -0.90 | -1.23 |
| 149 | Y340-1 | 20.00 | 0.95 | 7.22 | 10.25 | 6.61 | 0.42 |
| 150 | Y340-2 | 12.00 | 1.71 | 7.73 | 4.72 | 3.53 | -0.39 |
| 151 | Y340-3 | 5.00 | 0.98 | 2.41 | 2.16 | 1.45 | -0.10 |
| 152 | Y340-4 | 24.00 | 1.40 | 12.07 | 14.11 | 7.61 | 0.17 |
| 153 | Y340-5 | 0.10 | 0.64 | 0.49 | 0.36 | -0.24 | -0.26 |
| 154 | Y340S | 0.15 | 0.81 | 0.50 | 0.31 | -0.38 | -0.39 |
| 155 | Y340S-1 | 8.00 | 1.97 | 4.17 | 2.09 | 1.12 | -0.50 |
| 156 | Y340S-2 | 18.00 | 0.17 | 1.46 | 3.10 | 7.62 | 1.12 |
| 157 | Y340S-3 | 0.50 | 1.50 | 0.19 | 0.02 | -0.87 | -0.91 |
| 158 | Y340S-4 | 16.00 | 1.94 | 9.92 | 6.15 | 4.11 | -0.38 |
| 159 | Y340W | 1.50 | 0.58 | 0.69 | 0.67 | 0.18 | -0.03 |
| 160 | Y340W-1 | 1.80 | 0.59 | 0.79 | 0.78 | 0.34 | -0.01 |
| 161 | Y340W-2 | 0.40 | 0.37 | 0.36 | 0.35 | -0.02 | -0.04 |
| 162 | Y340W-3 | 2.00 | 0.72 | 0.97 | 0.87 | 0.34 | -0.10 |
| 163 | Y340W-4 | 0.10 | 0.31 | 0.28 | 0.25 | -0.09 | -0.11 |
| 164 | S251E-1 | 0.82 | 0.72 | 0.66 | 0.55 | -0.09 | -0.16 |
| 165 | S266 | 6.25 | 1.89 | 4.18 | 0.50 | 1.21 | -0.88 |
| 166 | S266-2 | 0.55 | 0.89 | 0.56 | 0.32 | -0.37 | -0.42 |
| 167 | S266-3 | 7.63 | 1.30 | 3.05 | 2.61 | 1.34 | -0.14 |
| 168 | Y250-1 | 1.75 | 0.82 | 0.93 | 0.76 | 0.13 | -0.19 |
| 169 | Y250-2 | 3.63 | 1.14 | 1.95 | 1.27 | 0.71 | -0.35 |
| 170 | Y250-3 | 9.58 | 1.96 | 5.34 | 2.65 | 1.73 | -0.50 |
| 171 | Y286 | 4.82 | 0.51 | 1.20 | 1.48 | 1.35 | 0.24 |
| 172 | Y286-1 | 0.28 | 0.37 | 0.35 | 0.32 | -0.07 | -0.08 |
| 173 | Y286-2 | 0.52 | 0.72 | 0.57 | 0.43 | -0.20 | -0.24 |
| 174 | Y288-1 | 5.20 | 0.90 | 1.80 | 1.79 | 1.01 | -0.01 |
| 175 | Y288-4 | 8.00 | 0.55 | 2.12 | 2.99 | 2.86 | 0.41 |
| 176 | Y289 | 43.06 | 0.00 | 0.00 | 0.00 | 12.99 | 1.27 |
| 177 | Y289-3 | 35.76 | 2.48 | 25.76 | 16.74 | 9.40 | -0.35 |
| 178 | Y325S | 1.30 | 0.07 | 0.10 | 0.14 | 0.58 | 0.35 |
| 179 | S23-1 | 1.00 | 0.43 | 0.49 | 0.50 | 0.13 | 0.01 |
| 180 | S60 | 1.70 | 0.49 | 0.66 | 0.69 | 0.34 | 0.05 |
| 181 | S60-1 | 14.80 | 1.15 | 8.10 | 9.65 | 6.06 | 0.19 |
| 182 | S22 | 4.40 | 0.80 | 1.63 | 1.66 | 1.05 | 0.02 |
| 183 | S22-1 | 0.40 | 0.51 | 0.43 | 0.34 | -0.16 | -0.19 |
| 184 | S22-2 | 35.90 | 1.65 | 25.65 | 32.02 | 14.56 | 0.25 |
| 185 | S22-3 | 0.24 | 0.65 | 0.47 | 0.33 | -0.28 | -0.29 |
| 186 | S24-1 | 15.90 | 1.03 | 6.68 | 8.54 | 5.47 | 0.28 |
| 187 | S25-1 | 5.90 | 1.15 | 3.14 | 2.50 | 1.74 | -0.20 |
| 188 | S25-2 | 0.65 | 0.37 | 0.40 | 0.41 | 0.08 | 0.01 |
| 189 | S27-1 | 18.00 | 0.85 | 7.35 | 11.61 | 7.68 | 0.58 |
| 190 | S27-2 | 1.50 | 0.69 | 0.79 | 0.70 | 0.15 | -0.12 |
| 191 | S28-1 | 25.50 | 0.74 | 5.36 | 8.03 | 6.28 | 0.50 |
| 192 | S28-2 | 5.00 | 1.10 | 2.59 | 2.04 | 1.36 | -0.21 |
| 193 | S28W | 1.10 | 0.49 | 0.58 | 0.57 | 0.17 | -0.02 |
| 194 | S28W-1 | 1.10 | 0.52 | 0.60 | 0.57 | 0.16 | -0.04 |
| 195 | S28W-2 | 1.30 | 0.21 | 0.31 | 0.38 | 0.46 | 0.23 |
| 196 | S36-1 | 4.40 | 0.97 | 1.78 | 1.62 | 0.84 | -0.09 |
| 197 | S37-1 | 1.43 | 0.98 | 0.85 | 0.48 | -0.13 | -0.44 |
| 198 | S37-2 | 2.60 | 1.12 | 1.36 | 0.91 | 0.21 | -0.33 |
| 199 | S38-1 | 1.10 | 0.90 | 0.78 | 0.57 | -0.14 | -0.27 |
| 200 | S38-2 | 10.10 | 0.94 | 3.12 | 3.48 | 2.30 | 0.12 |
| 201 | S38-3 | 11.50 | 1.63 | 6.92 | 4.66 | 3.25 | -0.33 |
| 202 | S4-1 | 26.00 | 3.22 | 29.41 | -8.96 | 8.13 | -1.30 |
| 203 | S41-1 | 6.30 | 0.67 | 1.88 | 2.26 | 1.82 | 0.20 |
| 204 | S41-2 | 3.68 | 0.57 | 1.28 | 1.52 | 1.26 | 0.18 |
| 205 | S41-3 | 0.20 | 0.26 | 0.25 | 0.25 | -0.01 | -0.01 |
| 206 | S4-2 | 6.50 | 0.54 | 1.76 | 2.37 | 2.26 | 0.35 |
| 207 | S42-1 | 2.58 | 0.97 | 1.23 | 1.00 | 0.26 | -0.19 |
| 208 | S42-2 | 12.88 | 0.89 | 3.39 | 4.04 | 2.80 | 0.19 |
| 209 | S42E | 52.00 | 3.27 | 70.16 | 2.84 | 20.47 | -0.96 |
| 210 | S47-1 | 1.00 | 1.48 | 0.59 | 0.16 | -0.60 | -0.73 |
| 211 | S47N | 3.50 | 0.38 | 0.92 | 1.26 | 1.44 | 0.36 |
| 212 | S47N-1 | 2.20 | 0.76 | 1.02 | 0.91 | 0.34 | -0.10 |
| 213 | S47N-2 | 3.20 | 0.88 | 1.55 | 1.32 | 0.76 | -0.14 |
| 214 | S47W | 5.50 | 1.43 | 2.92 | 1.76 | 1.04 | -0.40 |
| 215 | S47W-1 | 5.10 | 0.78 | 2.05 | 2.23 | 1.65 | 0.09 |
| 216 | S47W-2 | 5.50 | 1.31 | 3.03 | 1.95 | 1.31 | -0.36 |
| 217 | S47W-3 | 32.90 | 2.16 | 26.38 | 19.95 | 11.23 | -0.24 |
| 218 | S48-1 | 0.20 | 1.09 | 0.45 | 0.18 | -0.58 | -0.59 |
| 219 | S49 | 0.45 | 0.26 | 0.28 | 0.29 | 0.08 | 0.05 |
| 220 | S49-1 | 1.13 | 0.27 | 0.38 | 0.46 | 0.42 | 0.20 |
| 221 | S50 | 10.00 | 0.64 | 3.02 | 4.32 | 3.72 | 0.43 |
| 222 | S50-1 | 2.00 | 0.67 | 0.94 | 0.88 | 0.41 | -0.06 |
| 223 | S50-2 | 2.00 | 0.96 | 1.06 | 0.82 | 0.09 | -0.22 |
| 224 | S50E | 18.30 | 1.22 | 9.69 | 11.79 | 6.95 | 0.22 |
| 225 | S50E-1 | 17.50 | 1.58 | 8.24 | 7.53 | 4.21 | -0.09 |
| 226 | S50E-2 | 30.00 | 1.19 | 9.49 | 12.42 | 6.99 | 0.31 |
| 227 | S50E-3 | 1.60 | 1.62 | 0.63 | 0.03 | -0.61 | -0.95 |
| 228 | S51 | 0.40 | 1.07 | 0.43 | 0.17 | -0.59 | -0.62 |
| 229 | S51-1 | 0.50 | 0.43 | 0.42 | 0.38 | -0.04 | -0.08 |
| 230 | S56-2 | 4.10 | 0.35 | 0.91 | 1.27 | 1.59 | 0.40 |
| 231 | Y154-1 | 2.70 | 0.53 | 0.85 | 0.93 | 0.60 | 0.09 |
| 232 | Y175 | 4.00 | 0.93 | 1.84 | 1.63 | 0.98 | -0.12 |
| 233 | Y175-1 | 5.70 | 1.09 | 2.56 | 2.23 | 1.35 | -0.13 |
| 234 | Y175-2 | 2.20 | 1.67 | 1.16 | 0.34 | -0.31 | -0.71 |
| 235 | Y175S | 1.50 | 0.29 | 0.44 | 0.54 | 0.54 | 0.23 |
| 236 | Y175S-1 | 1.20 | 0.27 | 0.33 | 0.37 | 0.25 | 0.12 |
| 237 | Y175S-2 | 1.50 | 0.51 | 0.66 | 0.68 | 0.30 | 0.02 |
| 238 | Y175S-4 | 2.20 | 0.31 | 0.49 | 0.59 | 0.58 | 0.20 |
| 239 | Y177 | 1.00 | 0.24 | 0.33 | 0.39 | 0.38 | 0.20 |
| 240 | Y177-1 | 0.10 | 0.24 | 0.24 | 0.22 | -0.04 | -0.05 |
| 241 | Y177W | 1.00 | 0.23 | 0.31 | 0.37 | 0.36 | 0.20 |
| 242 | Y185-1 | 2.20 | 0.28 | 0.51 | 0.66 | 0.83 | 0.31 |
| 243 | Y201 | 0.60 | 0.62 | 0.54 | 0.44 | -0.13 | -0.18 |
| 244 | Y201-1 | 30.00 | 0.66 | 8.66 | 17.05 | 12.11 | 0.97 |
| 245 | Y201-2 | 1.00 | 0.59 | 0.62 | 0.54 | 0.03 | -0.12 |
| 246 | Y201-3 | 2.20 | 0.66 | 0.94 | 0.92 | 0.44 | -0.02 |
| 247 | Y201S-1 | 1.00 | 0.33 | 0.42 | 0.46 | 0.27 | 0.11 |
| 248 | Y201S-2 | 3.00 | 0.27 | 0.48 | 0.60 | 0.80 | 0.26 |
| 249 | Y201S-3 | 5.50 | 0.75 | 1.56 | 1.68 | 1.07 | 0.08 |
| 250 | Y252S | 3.40 | 1.78 | 1.80 | 0.86 | 0.01 | -0.52 |
| 251 | Y252S-1 | 2.50 | 0.73 | 1.00 | 0.95 | 0.36 | -0.04 |
| 252 | Y272S | 30.00 | 0.41 | 4.76 | 9.62 | 10.63 | 1.02 |
| 253 | Y272S-1 | 0.80 | 1.27 | 0.65 | 0.28 | -0.49 | -0.57 |
| 254 | Y272S-2 | 30.00 | 0.00 | 0.00 | 0.00 | 14.56 | 1.77 |
| 255 | Y272SE | 1.00 | 0.59 | 0.61 | 0.55 | 0.04 | -0.10 |
| 256 | Y310E | 0.90 | 0.35 | 0.43 | 0.47 | 0.24 | 0.08 |
| 257 | Y311 | 4.30 | 1.03 | 1.99 | 1.66 | 0.93 | -0.16 |
| 258 | Y311E | 20.00 | 1.71 | 9.15 | 8.20 | 4.34 | -0.10 |
| 259 | Y311E-1 | 2.50 | 0.81 | 1.24 | 1.06 | 0.53 | -0.15 |
| 260 | Y311E-2 | 3.50 | 1.54 | 2.02 | 0.43 | 0.31 | -0.78 |
| 261 | Y311E-3 | 13.00 | 1.72 | 6.43 | 4.77 | 2.73 | -0.26 |
| 262 | Y311E-4 | 1.20 | 1.72 | 0.19 | -0.02 | -0.89 | -1.11 |
| 263 | Y338W | 13.00 | 0.65 | 3.33 | 4.79 | 4.10 | 0.44 |
| 264 | Y338W-1 | 50.00 | 3.11 | 49.81 | 17.69 | 15.01 | -0.64 |
| 265 | Y338W-2 | 18.00 | 2.42 | 12.18 | 4.87 | 4.04 | -0.60 |
| 266 | Y339 | 3.10 | 0.09 | 0.16 | 0.22 | 0.84 | 0.33 |
| 267 | Y341 | 0.60 | 0.53 | 0.50 | 0.46 | -0.04 | -0.08 |
| 268 | Y341-3 | 1.70 | 0.61 | 0.78 | 0.76 | 0.29 | -0.03 |
| 269 | Y345 | 0.50 | 0.38 | 0.39 | 0.38 | 0.00 | -0.02 |
| 270 | Y345-1 | 5.70 | 0.59 | 1.69 | 2.12 | 1.86 | 0.26 |
| 271 | YP2 | 30.00 | 2.62 | 28.47 | 9.16 | 9.87 | -0.68 |
| 272 | S212-1 | 0.64 | 0.01 | 0.01 | 0.02 | 0.25 | 0.21 |
| 273 | S214-1 | 1.74 | 0.61 | 0.84 | 0.81 | 0.39 | -0.04 |
| 274 | S232-1 | 4.99 | 1.28 | 2.43 | 1.74 | 0.91 | -0.29 |
| 275 | S248-4 | 8.00 | 0.14 | 0.53 | 0.87 | 2.73 | 0.63 |
| 276 | Y161-1 | 0.40 | 0.68 | 0.56 | 0.46 | -0.17 | -0.18 |
| 277 | S228 | 8.34 | 1.71 | 5.81 | 2.21 | 2.40 | -0.62 |
| 278 | S228-1 | 10.98 | 1.25 | 4.29 | 4.15 | 2.43 | -0.03 |
| 279 | S229-1 | 5.37 | 1.01 | 2.01 | 1.88 | 1.00 | -0.07 |
| 280 | S229-2 | 10.03 | 1.31 | 6.01 | 5.09 | 3.60 | -0.15 |
| 281 | S229W | 6.31 | 1.49 | 3.42 | 2.04 | 1.29 | -0.40 |
| 282 | S229W-2 | 2.00 | 0.25 | 0.46 | 0.62 | 0.87 | 0.35 |
| 283 | S231-1 | 0.30 | 0.22 | 0.23 | 0.24 | 0.05 | 0.04 |
| 284 | S231-2 | 3.19 | 0.60 | 1.25 | 1.40 | 1.07 | 0.12 |
| 285 | S231-3 | 10.17 | 1.86 | 7.59 | 2.49 | 3.07 | -0.67 |
| 286 | S251 | 0.74 | 0.61 | 0.56 | 0.45 | -0.09 | -0.19 |
| 287 | S251E | 15.82 | 3.06 | 13.88 | -3.35 | 3.54 | -1.24 |
| 288 | S254 | 58.99 | 3.21 | 69.71 | 20.84 | 20.70 | -0.70 |
| 289 | S254-1 | 20.10 | 2.06 | 10.24 | 7.43 | 3.96 | -0.27 |
| 290 | S254-2 | 2.00 | 1.02 | 1.11 | 0.72 | 0.08 | -0.35 |
| 291 | S255 | 21.06 | 2.75 | 16.08 | 3.83 | 4.85 | -0.76 |
| 292 | S255-1 | 5.50 | 2.41 | 3.28 | -1.08 | 0.36 | -1.33 |
| 293 | S255-2 | 0.40 | 1.06 | 0.44 | 0.17 | -0.58 | -0.61 |
| 294 | S256E | 2.60 | 0.85 | 1.30 | 1.08 | 0.53 | -0.17 |
| 295 | S256E-1 | 1.00 | 0.84 | 0.67 | 0.42 | -0.20 | -0.38 |
| 296 | S256E-2 | 2.75 | 0.97 | 1.29 | 1.05 | 0.32 | -0.18 |
| 297 | S256E-3 | 0.30 | 0.72 | 0.53 | 0.38 | -0.26 | -0.27 |
| 298 | S256E-4 | 38.16 | 1.59 | 24.09 | 31.60 | 14.14 | 0.31 |
| 299 | S256W | 1.80 | 0.49 | 0.68 | 0.71 | 0.37 | 0.06 |
| 300 | S256W-1 | 6.00 | 1.58 | 3.91 | 1.40 | 1.47 | -0.64 |
| 301 | S256W-2 | 15.00 | 1.47 | 10.01 | 8.88 | 5.83 | -0.11 |
| 302 | S256W-3 | 7.00 | 1.42 | 3.11 | 2.37 | 1.20 | -0.24 |
| 303 | S256W-5 | 5.00 | 0.60 | 1.31 | 1.52 | 1.18 | 0.16 |
| 304 | S257 | 6.89 | 1.27 | 3.62 | 2.78 | 1.86 | -0.23 |
| 305 | S257-1 | 0.26 | 0.86 | 0.58 | 0.38 | -0.33 | -0.34 |
| 306 | S257-2 | 5.10 | 1.00 | 1.86 | 1.74 | 0.86 | -0.07 |
| 307 | S257-3 | 3.60 | 1.27 | 1.78 | 1.20 | 0.41 | -0.33 |
| 308 | S257E | 5.50 | 0.55 | 1.77 | 2.38 | 2.22 | 0.34 |
| 309 | S257E-1 | 8.50 | 0.81 | 3.29 | 4.03 | 3.03 | 0.23 |
| 310 | S257E-2 | 5.85 | 0.73 | 1.69 | 1.87 | 1.30 | 0.11 |
| 311 | S257E-3 | 0.10 | 0.66 | 0.43 | 0.28 | -0.35 | -0.36 |
| 312 | S264 | 1.79 | 0.86 | 0.99 | 0.72 | 0.15 | -0.27 |
| 313 | S264-1 | 2.43 | 0.96 | 1.23 | 0.94 | 0.28 | -0.24 |
| 314 | S264-2 | 11.95 | 0.91 | 4.71 | 5.99 | 4.20 | 0.27 |
| 315 | S265 | 22.59 | 1.86 | 12.57 | 10.47 | 5.75 | -0.17 |
| 316 | S265-1 | 6.99 | 1.24 | 3.95 | 3.04 | 2.20 | -0.23 |
| 317 | S265-2 | 8.22 | 1.26 | 3.50 | 3.07 | 1.78 | -0.12 |
| 318 | S266-1 | 16.62 | 1.51 | 11.26 | 10.11 | 6.44 | -0.10 |
| 319 | S267 | 43.80 | 3.18 | 35.68 | 13.76 | 10.23 | -0.61 |
| 320 | S267-1 | 8.18 | 1.22 | 4.32 | 3.72 | 2.55 | -0.14 |
| 321 | S267-2 | 10.87 | 2.14 | 7.00 | 2.41 | 2.27 | -0.66 |
| 322 | S267-3 | 11.50 | 2.82 | 8.55 | -1.00 | 2.04 | -1.12 |
| 323 | Y119 | 10.26 | 0.85 | 2.82 | 3.29 | 2.32 | 0.17 |
| 324 | Y121 | 2.02 | 0.76 | 1.03 | 0.87 | 0.36 | -0.15 |
| 325 | Y126 | 1.18 | 0.39 | 0.51 | 0.55 | 0.30 | 0.08 |
| 326 | Y126S | 2.00 | 0.43 | 0.71 | 0.82 | 0.65 | 0.16 |
| 327 | Y126S-1 | 1.20 | 0.25 | 0.33 | 0.37 | 0.29 | 0.14 |
| 328 | Y126S-2 | 1.20 | 0.57 | 0.64 | 0.60 | 0.11 | -0.06 |
| 329 | Y169-1 | 16.92 | 2.40 | 15.56 | 1.52 | 5.48 | -0.90 |
| 330 | Y169-2 | 39.83 | 3.49 | 41.61 | -0.62 | 10.92 | -1.01 |
| 331 | Y216E | 0.77 | 0.33 | 0.37 | 0.39 | 0.11 | 0.04 |
| 332 | Y250-4 | 19.05 | 2.05 | 12.87 | 7.69 | 5.29 | -0.40 |
| 333 | Y251 | 3.17 | 0.94 | 1.43 | 1.21 | 0.51 | -0.15 |
| 334 | Y251-2 | 6.82 | 0.76 | 2.11 | 2.42 | 1.77 | 0.14 |
| 335 | Y251-3 | 6.80 | 1.03 | 3.29 | 3.16 | 2.21 | -0.04 |
| 336 | Y251E | 15.00 | 1.46 | 6.34 | 5.96 | 3.35 | -0.06 |
| 337 | Y270 | 43.98 | 2.29 | 33.19 | 28.41 | 13.50 | -0.14 |
| 338 | Y270-1 | 4.95 | 0.57 | 1.36 | 1.65 | 1.41 | 0.21 |
| 339 | Y270-2 | 3.68 | 0.51 | 1.20 | 1.50 | 1.36 | 0.25 |
| 340 | Y270-3 | 0.18 | 0.38 | 0.33 | 0.28 | -0.14 | -0.15 |
| 341 | Y270W | 0.50 | 0.20 | 0.23 | 0.25 | 0.13 | 0.10 |
| 342 | Y270W-1 | 0.50 | 0.32 | 0.33 | 0.34 | 0.04 | 0.01 |
| 343 | Y270W-2 | 1.20 | 0.32 | 0.43 | 0.50 | 0.36 | 0.15 |
| 344 | Y270W-3 | 1.20 | 0.24 | 0.32 | 0.37 | 0.31 | 0.16 |
| 345 | Y270W-4 | 3.50 | 0.33 | 0.62 | 0.78 | 0.88 | 0.24 |
| 346 | Y271 | 4.06 | 0.39 | 0.92 | 1.21 | 1.36 | 0.32 |
| 347 | Y271N | 1.20 | 0.37 | 0.50 | 0.55 | 0.36 | 0.12 |
| 348 | Y271N-1 | 6.10 | 0.65 | 1.74 | 2.08 | 1.67 | 0.20 |
| 349 | Y271N-2 | 1.10 | 0.32 | 0.42 | 0.48 | 0.33 | 0.14 |
| 350 | Y271N-3 | 1.80 | 0.33 | 0.55 | 0.67 | 0.65 | 0.23 |
| 351 | Y271N-4 | 2.50 | 0.32 | 0.60 | 0.78 | 0.90 | 0.29 |
| 352 | Y271S | 1.26 | 0.41 | 0.49 | 0.52 | 0.21 | 0.05 |
| 353 | Y272 | 48.97 | 1.89 | 23.16 | 26.49 | 11.23 | 0.14 |
| 354 | Y272-1 | 37.24 | 2.07 | 28.17 | 25.48 | 12.60 | -0.10 |
| 355 | Y272-2 | 1.52 | 1.05 | 0.93 | 0.59 | -0.11 | -0.36 |
| 356 | Y272-3 | 0.08 | 1.45 | 0.07 | 0.00 | -0.95 | -0.97 |
| 357 | Y272-4 | 6.50 | 1.87 | 4.45 | 0.59 | 1.37 | -0.87 |
| 358 | Y272E | 1.89 | 0.90 | 0.99 | 0.80 | 0.11 | -0.19 |
| 359 | Y272E-1 | 0.20 | 0.00 | 0.00 | 0.00 | 0.17 | 0.16 |
| 360 | Y272E-2 | 12.00 | 1.33 | 4.60 | 4.38 | 2.47 | -0.05 |
| 361 | Y285 | 10.02 | 1.71 | 4.86 | 3.34 | 1.84 | -0.31 |
| 362 | Y285-1 | 27.76 | 2.21 | 17.73 | 12.49 | 7.02 | -0.30 |
| 363 | Y285-2 | 14.56 | 2.38 | 12.54 | 1.01 | 4.28 | -0.92 |
| 364 | Y285-3 | 3.20 | 0.47 | 0.82 | 0.94 | 0.73 | 0.15 |
| 365 | Y286-3 | 2.10 | 0.25 | 0.47 | 0.62 | 0.86 | 0.34 |
| 366 | Y286W | 1.80 | 1.00 | 1.01 | 0.77 | 0.01 | -0.24 |
| 367 | Y286W-1 | 1.80 | 0.95 | 1.02 | 0.66 | 0.07 | -0.35 |
| 368 | Y286W-2 | 20.00 | 0.02 | 0.15 | 0.26 | 5.20 | 0.79 |
| 369 | Y286W-3 | 0.60 | 0.40 | 0.41 | 0.40 | 0.02 | -0.01 |
| 370 | Y288 | 10.69 | 1.66 | 5.65 | 3.88 | 2.40 | -0.31 |
| 371 | Y288-2 | 0.30 | 0.66 | 0.53 | 0.42 | -0.20 | -0.20 |
| 372 | Y288-3 | 14.30 | 1.26 | 7.14 | 7.50 | 4.66 | 0.05 |
| 373 | Y288W | 11.00 | 0.71 | 2.78 | 3.59 | 2.90 | 0.29 |
| 374 | Y288W-1 | 6.00 | 0.54 | 1.76 | 2.38 | 2.27 | 0.36 |
| 375 | Y288W-2 | 0.25 | 0.62 | 0.46 | 0.34 | -0.26 | -0.27 |
| 376 | Y288W-3 | 1.80 | 0.53 | 0.70 | 0.72 | 0.33 | 0.03 |
| 377 | Y289-1 | 15.16 | 0.00 | 0.00 | 0.00 | 4.92 | 0.89 |
| 378 | Y289-2 | 18.50 | 2.75 | 17.78 | -1.72 | 5.46 | -1.10 |
| 379 | Y301S | 0.79 | 0.76 | 0.62 | 0.45 | -0.18 | -0.27 |
| 380 | Y301S-2 | 0.21 | 0.51 | 0.38 | 0.29 | -0.24 | -0.25 |
| 381 | Y327-1 | 3.05 | 0.61 | 1.21 | 1.33 | 0.99 | 0.10 |
| 382 | Y329 | 2.93 | 0.53 | 0.94 | 1.06 | 0.77 | 0.13 |
| 383 | Y329-1 | 2.00 | 0.67 | 0.83 | 0.80 | 0.25 | -0.04 |
| 384 | Y329-2 | 5.00 | 0.87 | 2.16 | 2.14 | 1.47 | -0.01 |
| 385 | Y329-3 | 3.00 | 1.06 | 1.46 | 1.10 | 0.38 | -0.25 |
| 386 | Y329-4 | 5.30 | 0.88 | 1.84 | 1.85 | 1.09 | 0.00 |
| 387 | Y329-5 | 0.70 | 0.11 | 0.14 | 0.16 | 0.21 | 0.16 |
| 388 | Y329W | 1.00 | 0.50 | 0.56 | 0.54 | 0.12 | -0.04 |
| 389 | Y329W-1 | 2.80 | 0.30 | 0.49 | 0.59 | 0.64 | 0.21 |
| 390 | Y329W-2 | 0.50 | 0.47 | 0.44 | 0.40 | -0.06 | -0.10 |
| 391 | Y330 | 1.97 | 0.37 | 0.59 | 0.70 | 0.61 | 0.19 |
| 392 | Y330-1 | 1.69 | 0.51 | 0.67 | 0.70 | 0.32 | 0.03 |
| 393 | Y330-2 | 6.67 | 0.92 | 2.48 | 2.56 | 1.68 | 0.03 |
| 394 | Y331 | 1.02 | 0.32 | 0.38 | 0.41 | 0.19 | 0.08 |
| 395 | Y331E | 10.91 | 0.83 | 3.71 | 4.70 | 3.46 | 0.27 |
| 396 | Y331E-1 | 1.00 | 0.30 | 0.39 | 0.44 | 0.31 | 0.14 |
| 397 | Y331W | 28.00 | 1.24 | 12.33 | 16.75 | 8.98 | 0.36 |
| 398 | Y331W-2 | 2.40 | 0.78 | 1.15 | 1.01 | 0.48 | -0.12 |
| 399 | Y333 | 1.19 | 0.35 | 0.43 | 0.47 | 0.24 | 0.09 |
| 400 | Y333-1 | 0.51 | 0.56 | 0.50 | 0.42 | -0.11 | -0.15 |
| 401 | Y333N-1 | 15.01 | 0.54 | 2.85 | 4.28 | 4.27 | 0.50 |
| 402 | YP6 | 112.63 | 2.76 | 144.73 | 185.18 | 51.43 | 0.28 |
| 403 | YP7 | 3.34 | 1.25 | 1.61 | 1.16 | 0.28 | -0.28 |
| 404 | S26 | 1.23 | 0.23 | 0.28 | 0.33 | 0.22 | 0.17 |
| 405 | S252 | 1.15 | 0.48 | 0.51 | 0.51 | 0.06 | 0.00 |
| 406 | S253 | 3.02 | 0.56 | 0.87 | 0.99 | 0.57 | 0.14 |
| 407 | S253E | 16.40 | 0.56 | 3.45 | 6.26 | 5.13 | 0.81 |
| 408 | S253E-1 | 0.10 | 0.01 | 0.01 | 0.01 | 0.14 | 0.14 |
| 409 | S256 | 12.81 | 1.69 | 4.56 | 3.44 | 1.70 | -0.25 |
| 410 | S256-1 | 16.05 | 2.31 | 8.10 | 2.59 | 2.51 | -0.68 |
| 411 | S256-2 | 0.11 | 1.45 | 0.32 | 0.07 | -0.78 | -0.78 |
| 412 | S256W-4 | 2.00 | 0.62 | 0.73 | 0.72 | 0.18 | -0.02 |
| 413 | Y124 | 0.33 | 0.40 | 0.36 | 0.33 | -0.10 | -0.08 |
| 414 | Y124-1 | 0.41 | 0.40 | 0.37 | 0.35 | -0.07 | -0.05 |
| 415 | Y218 | 15.58 | 0.63 | 3.53 | 5.96 | 4.58 | 0.69 |
| 416 | Y251-1 | 3.63 | 0.61 | 0.94 | 1.03 | 0.53 | 0.10 |
| 417 | Y251E-1 | 54.00 | 3.67 | 32.95 | 8.66 | 7.99 | -0.74 |
| 418 | Y251E-2 | 17.00 | 3.13 | 8.39 | 0.11 | 1.68 | -0.99 |
| 419 | Y251S | 19.00 | 1.89 | 9.14 | 6.20 | 3.83 | -0.32 |
| 420 | Y251S-1 | 2.10 | 0.57 | 0.71 | 0.74 | 0.25 | 0.04 |
| 421 | Y269E | 0.40 | 0.85 | 0.38 | 0.18 | -0.56 | -0.53 |
| 422 | Y269E-1 | 1.30 | 0.38 | 0.44 | 0.49 | 0.17 | 0.10 |
| 423 | Y269E-5 | 1.20 | 0.50 | 0.52 | 0.52 | 0.06 | -0.01 |
| 424 | Y271-1 | 6.56 | 0.25 | 0.62 | 0.91 | 1.42 | 0.47 |
| 425 | Y272N | 49.00 | 0.00 | 0.00 | 0.00 | 9.09 | 1.15 |
| 426 | Y272N-1 | 21.00 | 2.83 | 14.57 | -2.32 | 4.15 | -1.16 |
| 427 | Y272N-2 | 32.00 | 2.96 | 26.58 | -2.38 | 7.97 | -1.09 |
| 428 | Y272N-3 | 16.00 | 2.35 | 8.47 | 2.10 | 2.60 | -0.75 |
| 429 | Y272N-4 | 10.10 | 2.15 | 4.08 | 1.65 | 0.90 | -0.60 |
| 430 | Y287 | 1.52 | 0.51 | 0.58 | 0.59 | 0.12 | 0.02 |
| 431 | Y287-1 | 1.20 | 0.52 | 0.54 | 0.53 | 0.04 | -0.03 |
| 432 | Y287-2 | 38.00 | 0.41 | 4.84 | 11.12 | 10.77 | 1.30 |
| 433 | Y304 | 20.18 | 2.07 | 12.19 | 5.75 | 4.89 | -0.53 |
| 434 | Y304S | 16.20 | 0.32 | 1.23 | 1.92 | 2.85 | 0.56 |
| 435 | Y304S-1 | 1.10 | 0.53 | 0.53 | 0.51 | 0.00 | -0.04 |
| 436 | Y304S-2 | 15.00 | 1.43 | 4.67 | 4.46 | 2.27 | -0.04 |
| 437 | Y304S-3 | 3.00 | 0.23 | 0.35 | 0.45 | 0.57 | 0.28 |
| 438 | Y304W | 1.10 | 0.37 | 0.42 | 0.45 | 0.13 | 0.08 |
| 439 | Y304W-1 | 30.00 | 1.32 | 11.38 | 15.79 | 7.64 | 0.39 |
| 440 | Y304W-3 | 1.80 | 0.80 | 0.79 | 0.66 | -0.02 | -0.17 |
| 441 | Y327 | 1.60 | 0.20 | 0.26 | 0.31 | 0.28 | 0.19 |
| 442 | Y331E-2 | 0.50 | 0.50 | 0.45 | 0.41 | -0.10 | -0.09 |
| 443 | Y332-1 | 31.94 | 1.53 | 10.60 | 12.55 | 5.92 | 0.18 |
| 444 | Y332-2 | 5.10 | 1.20 | 1.95 | 1.40 | 0.62 | -0.28 |
| 445 | Y332-3 | 10.00 | 1.62 | 4.20 | 2.61 | 1.59 | -0.38 |
| 446 | Y332-4 | 0.50 | 0.90 | 0.53 | 0.32 | -0.41 | -0.40 |
| 447 | Y332S | 20.00 | 1.58 | 9.96 | 9.35 | 5.30 | -0.06 |
| 448 | Y332S-1 | 18.00 | 0.75 | 3.10 | 4.32 | 3.15 | 0.39 |
| 449 | Y333-2 | 1.02 | 0.17 | 0.21 | 0.24 | 0.20 | 0.17 |
| 450 | Y333N | 2.60 | 0.53 | 0.67 | 0.72 | 0.27 | 0.07 |
| 451 | YP8 | 10.08 | 2.38 | 4.75 | 0.15 | 1.00 | -0.97 |
| 452 | Y340E | 13.50 | 2.01 | 6.32 | 2.90 | 2.15 | -0.54 |
| 453 | S27S | 70.00 | 2.71 | 56.80 | 46.42 | 19.95 | -0.18 |
| 454 | S27S-2 | 50.00 | 1.52 | 18.55 | 27.29 | 11.19 | 0.47 |
| 455 | Y338S-3 | 1.20 | 0.39 | 0.43 | 0.45 | 0.09 | 0.05 |
| 456 | Y338S-5 | 10.00 | 1.65 | 3.92 | 2.54 | 1.38 | -0.35 |
| 457 | Y338W-3 | 12.00 | 2.27 | 4.96 | 1.97 | 1.19 | -0.60 |
| 458 | Y340E-2 | 0.90 | 0.76 | 0.56 | 0.40 | -0.26 | -0.28 |
| 459 | Y340E-3 | 6.50 | 2.26 | 2.57 | -0.70 | 0.14 | -1.27 |
| 460 | A2-1 | 0.10 | 0.04 | 0.04 | 0.05 | 0.11 | 0.10 |
| 461 | A2-4 | 0.20 | 0.00 | 0.00 | 0.00 | 0.18 | 0.20 |
| 462 | Y383-1 | 0.50 | 0.21 | 0.23 | 0.26 | 0.10 | 0.12 |
| 463 | Y383-2 | 1.00 | 0.24 | 0.29 | 0.33 | 0.19 | 0.16 |
| 464 | Y383-3 | 20.00 | 1.35 | 7.24 | 8.15 | 4.36 | 0.13 |
| 465 | Y338E | 1.00 | 0.62 | 0.54 | 0.46 | -0.12 | -0.16 |
| 466 | Y338E-1 | 1.10 | 0.61 | 0.57 | 0.51 | -0.07 | -0.11 |
| 467 | Y338E-2 | 1.30 | 0.61 | 0.61 | 0.57 | -0.01 | -0.07 |
| 468 | A1 | 0.20 | 0.06 | 0.07 | 0.08 | 0.12 | 0.13 |
| 469 | A10 | 1.20 | 0.30 | 0.34 | 0.37 | 0.13 | 0.09 |
| 470 | A10-1 | 0.50 | 0.52 | 0.42 | 0.36 | -0.18 | -0.16 |
| 471 | A10-2 | 30.00 | 2.11 | 18.14 | 12.79 | 7.58 | -0.30 |
| 472 | A10-3 | 40.00 | 0.00 | 0.00 | 0.00 | 7.21 | 1.03 |
| 473 | A11 | 3.50 | 1.08 | 1.36 | 0.95 | 0.26 | -0.30 |
| 474 | A1-1 | 0.20 | 0.04 | 0.04 | 0.04 | 0.09 | 0.10 |
| 475 | A11-2 | 2.00 | 0.78 | 0.82 | 0.70 | 0.05 | -0.15 |
| 476 | A11-3 | 4.00 | 1.18 | 1.49 | 1.06 | 0.27 | -0.29 |
| 477 | A11P1 | 59.00 | 0.00 | 0.00 | 0.00 | 17.64 | 2.01 |
| 478 | A12 | 6.00 | 1.01 | 1.98 | 1.87 | 0.97 | -0.06 |
| 479 | A1-2 | 15.00 | 1.30 | 5.29 | 5.48 | 3.06 | 0.04 |
| 480 | A12-1 | 13.00 | 1.59 | 5.87 | 4.24 | 2.69 | -0.28 |
| 481 | A12-2 | 1.20 | 0.58 | 0.57 | 0.53 | -0.01 | -0.08 |
| 482 | A13 | 10.00 | 1.39 | 3.53 | 2.94 | 1.54 | -0.17 |
| 483 | A13-1 | 12.00 | 2.00 | 5.06 | 2.49 | 1.53 | -0.51 |
| 484 | A13-3 | 15.56 | 1.94 | 8.67 | 3.94 | 3.48 | -0.55 |
| 485 | A16 | 30.00 | 1.30 | 11.12 | 15.58 | 7.58 | 0.40 |
| 486 | A16-1 | 1.20 | 0.32 | 0.39 | 0.45 | 0.22 | 0.15 |
| 487 | A16-2 | 1.10 | 0.27 | 0.32 | 0.37 | 0.20 | 0.15 |
| 488 | A2 | 22.42 | 1.94 | 12.72 | 8.57 | 5.56 | -0.33 |
| 489 | A2-2 | 0.25 | 0.09 | 0.10 | 0.12 | 0.14 | 0.16 |
| 490 | A3 | 1.20 | 0.91 | 0.62 | 0.38 | -0.31 | -0.39 |
| 491 | A3-1 | 1.30 | 0.21 | 0.27 | 0.33 | 0.28 | 0.21 |
| 492 | A3-2 | 0.60 | 0.20 | 0.23 | 0.25 | 0.12 | 0.12 |
| 493 | A4 | 10.00 | 1.09 | 3.31 | 3.46 | 2.03 | 0.04 |
| 494 | A4-1 | 8.00 | 0.88 | 2.29 | 2.60 | 1.61 | 0.13 |
| 495 | A4-2 | 10.00 | 1.52 | 3.48 | 2.68 | 1.29 | -0.23 |
| 496 | A4-4 | 0.16 | 0.35 | 0.31 | 0.27 | -0.14 | -0.13 |
| 497 | A4P1 | 30.58 | 1.74 | 11.62 | 12.05 | 5.67 | 0.04 |
| 498 | A5 | 0.20 | 0.05 | 0.06 | 0.06 | 0.12 | 0.13 |
| 499 | A5-1 | 0.30 | 0.09 | 0.10 | 0.11 | 0.11 | 0.12 |
| 500 | A5-3 | 1.00 | 0.79 | 0.58 | 0.40 | -0.27 | -0.31 |
| 501 | A5-4 | 0.10 | 0.07 | 0.07 | 0.08 | 0.09 | 0.09 |
| 502 | A6 | 8.00 | 0.96 | 2.73 | 2.96 | 1.85 | 0.08 |
| 503 | A60 | 1.30 | 0.41 | 0.48 | 0.52 | 0.17 | 0.08 |
| 504 | A60-1 | 1.90 | 0.46 | 0.57 | 0.62 | 0.24 | 0.09 |
| 505 | A60-2 | 0.50 | 0.20 | 0.22 | 0.24 | 0.09 | 0.10 |
| 506 | A6-1 | 0.10 | 0.07 | 0.08 | 0.08 | 0.10 | 0.10 |
| 507 | A6-2 | 2.50 | 0.55 | 0.76 | 0.83 | 0.39 | 0.09 |
| 508 | A6-3 | 1.20 | 0.30 | 0.37 | 0.43 | 0.23 | 0.16 |
| 509 | A7-2 | 3.00 | 0.74 | 1.02 | 0.99 | 0.37 | -0.03 |
| 510 | A8 | 1.00 | 0.62 | 0.57 | 0.52 | -0.07 | -0.10 |
| 511 | A8-1 | 0.80 | 0.27 | 0.29 | 0.31 | 0.08 | 0.07 |
| 512 | A8-2 | 1.90 | 0.45 | 0.55 | 0.60 | 0.21 | 0.08 |
| 513 | A8-3 | 0.25 | 0.07 | 0.08 | 0.09 | 0.08 | 0.09 |
| 514 | A8-4 | 1.20 | 0.89 | 0.64 | 0.42 | -0.28 | -0.35 |
| 515 | Y145 | 26.21 | 2.20 | 15.55 | 8.68 | 6.06 | -0.44 |
| 516 | Y145-2 | 0.20 | 0.08 | 0.09 | 0.10 | 0.09 | 0.10 |
| 517 | Y145-3 | 0.30 | 0.13 | 0.14 | 0.15 | 0.09 | 0.11 |
| 518 | Y165 | 7.33 | 0.64 | 1.46 | 1.80 | 1.29 | 0.24 |
| 519 | Y165-1 | 0.60 | 0.18 | 0.21 | 0.23 | 0.12 | 0.12 |
| 520 | Y215-1 | 1.00 | 0.64 | 0.59 | 0.52 | -0.09 | -0.12 |
| 521 | Y215-2 | 10.00 | 1.41 | 4.42 | 3.28 | 2.13 | -0.26 |
| 522 | Y215-3 | 0.20 | 0.11 | 0.12 | 0.13 | 0.08 | 0.10 |
| 523 | Y294 | 3.28 | 0.48 | 0.80 | 0.97 | 0.67 | 0.21 |
| 524 | Y294-1 | 0.05 | 0.02 | 0.02 | 0.02 | 0.13 | 0.10 |
| 525 | Y294-2 | 1.00 | 0.20 | 0.25 | 0.29 | 0.22 | 0.19 |
| 526 | Y294-4 | 12.00 | 1.59 | 5.70 | 3.78 | 2.59 | -0.34 |
| 527 | Y382 | 2.66 | 0.57 | 0.80 | 0.87 | 0.41 | 0.08 |
| 528 | Y382-1 | 12.00 | 1.25 | 4.78 | 4.72 | 2.83 | -0.01 |
| 529 | Y382-2 | 15.59 | 1.73 | 8.03 | 5.28 | 3.64 | -0.34 |
| 530 | Y382-3 | 0.20 | 0.07 | 0.08 | 0.09 | 0.15 | 0.17 |
| 531 | Y382-4 | 15.00 | 1.53 | 4.45 | 4.06 | 1.92 | -0.09 |
| 532 | Y383 | 1.23 | 0.33 | 0.38 | 0.42 | 0.16 | 0.11 |
| 533 | Y383-4 | 1.20 | 0.29 | 0.34 | 0.37 | 0.15 | 0.11 |
| 534 | Y387 | 2.56 | 0.91 | 0.97 | 0.84 | 0.07 | -0.13 |
| 535 | Y387-1 | 0.30 | 0.13 | 0.15 | 0.17 | 0.12 | 0.14 |
| 536 | Y387-2 | 20.00 | 1.94 | 9.47 | 6.46 | 3.89 | -0.32 |
| 537 | Y387-3 | 0.20 | 0.46 | 0.39 | 0.34 | -0.14 | -0.13 |
| 538 | Y387P1 | 35.00 | 2.09 | 13.14 | 11.87 | 5.30 | -0.10 |
| 539 | Y388 | 1.10 | 0.32 | 0.38 | 0.44 | 0.21 | 0.15 |
| 540 | Y388-1 | 0.50 | 0.16 | 0.18 | 0.21 | 0.15 | 0.17 |
| 541 | Y388-2 | 0.15 | 0.08 | 0.09 | 0.10 | 0.10 | 0.11 |
| 542 | Y389 | 9.53 | 1.53 | 3.55 | 2.57 | 1.32 | -0.28 |
| 543 | Y389-2 | 1.20 | 1.28 | 0.47 | 0.14 | -0.63 | -0.70 |
| 544 | Y389-3 | 10.00 | 1.15 | 3.73 | 3.72 | 2.25 | 0.00 |
| 545 | A1-3 | 0.60 | 0.09 | 0.11 | 0.13 | 0.18 | 0.19 |
| 546 | A13-2 | 3.00 | 1.20 | 1.19 | 0.71 | -0.01 | -0.41 |
| 547 | A13P1 | 51.36 | 0.00 | 0.00 | 0.00 | 11.46 | 1.42 |
| 548 | A14 | 0.20 | 0.38 | 0.33 | 0.30 | -0.11 | -0.10 |
| 549 | A14-1 | 10.00 | 1.69 | 3.97 | 2.46 | 1.36 | -0.38 |
| 550 | A14-2 | 0.30 | 0.13 | 0.14 | 0.16 | 0.09 | 0.10 |
| 551 | A14-3 | 4.00 | 0.68 | 1.09 | 1.17 | 0.58 | 0.07 |
| 552 | A15-1 | 2.00 | 0.50 | 0.59 | 0.62 | 0.18 | 0.05 |
| 553 | A7 | 0.20 | 0.06 | 0.07 | 0.08 | 0.15 | 0.16 |
| 554 | A9-1 | 0.20 | 0.10 | 0.11 | 0.12 | 0.11 | 0.12 |
| 555 | A9-2 | 1.30 | 0.32 | 0.38 | 0.42 | 0.17 | 0.11 |
| 556 | A9P1 | 27.15 | 1.98 | 15.73 | 11.90 | 6.94 | -0.24 |
| 557 | Y294-3 | 32.00 | 1.45 | 8.99 | 10.79 | 5.18 | 0.20 |
| 558 | Y373 | 4.20 | 1.47 | 1.63 | 0.74 | 0.11 | -0.55 |
| 559 | Y373-1 | 7.00 | 0.80 | 1.77 | 2.01 | 1.22 | 0.13 |
| 560 | Y373-2 | 17.00 | 1.86 | 8.44 | 5.32 | 3.54 | -0.37 |
| 561 | Y373-3 | 0.80 | 0.29 | 0.32 | 0.35 | 0.10 | 0.09 |
| 562 | Y373-4 | 1.00 | 0.26 | 0.31 | 0.36 | 0.18 | 0.15 |
| 563 | S50N | 0.60 | 0.31 | 0.32 | 0.34 | 0.04 | 0.04 |
| 564 | S50N-1 | 1.50 | 0.43 | 0.53 | 0.58 | 0.22 | 0.09 |
| 565 | S50N-2 | 1.10 | 0.46 | 0.49 | 0.49 | 0.06 | 0.01 |
| 566 | Y338E-3 | 10.00 | 2.02 | 5.02 | 0.93 | 1.48 | -0.81 |
| 567 | A10P1 | 18.17 | 2.32 | 11.70 | 1.83 | 4.04 | -0.84 |
| 568 | Y340E-1 | 9.70 | 1.97 | 4.71 | 1.15 | 1.39 | -0.76 |
| 569 | Y389-4 | 4.00 | 1.49 | 1.54 | 0.77 | 0.03 | -0.50 |
| 570 | Y251E-3 | 1.50 | 1.80 | 0.06 | 0.00 | -0.97 | -1.08 |
| 571 | Y304W-2 | 13.67 | 2.21 | 6.95 | 1.87 | 2.14 | -0.73 |
| 572 | Y304W-4 | 0.65 | 0.46 | 0.42 | 0.39 | -0.08 | -0.08 |
| 573 | A9P2 | 27.15 | 0.91 | 8.58 | 15.27 | 8.40 | 0.78 |
| 574 | Y128 | 3.00 | 0.80 | 0.99 | 0.93 | 0.24 | -0.05 |
| 575 | Y144 | 7.35 | 0.70 | 1.59 | 1.91 | 1.27 | 0.20 |
| 576 | Y150 | 2.58 | 1.00 | 1.03 | 0.82 | 0.03 | -0.20 |
| 577 | S29 | 0.20 | 0.65 | 0.50 | 0.39 | -0.23 | -0.22 |
| 578 | S39-1 | 15.00 | 0.54 | 3.00 | 5.30 | 4.56 | 0.77 |
| 579 | S39-2 | 5.00 | 0.51 | 1.04 | 1.32 | 1.03 | 0.27 |
| 580 | S42E-2 | 5.00 | 1.45 | 1.97 | 1.01 | 0.35 | -0.49 |
| 581 | S51-3 | 5.00 | 0.37 | 0.69 | 0.90 | 0.88 | 0.29 |
| 582 | S51N-1 | 1.00 | 0.58 | 0.54 | 0.47 | -0.08 | -0.12 |
| 583 | S51N-3 | 4.00 | 0.00 | 0.00 | 0.00 | 1.32 | 0.68 |
| 584 | S61-1 | 60.00 | 0.00 | 0.00 | 0.00 | 17.02 | 1.92 |
| 585 | S61-2 | 50.00 | 2.85 | 42.29 | 16.07 | 13.84 | -0.62 |
| 586 | S8-1 | 1.00 | 0.87 | 0.63 | 0.43 | -0.28 | -0.31 |
| 587 | S8-2 | 6.00 | 2.54 | 2.11 | 0.26 | -0.17 | -0.88 |
| 588 | S8-3 | 1.50 | 1.09 | 0.73 | 0.42 | -0.33 | -0.43 |
| 589 | Y154-2 | 2.00 | 0.21 | 0.28 | 0.34 | 0.32 | 0.19 |
| 590 | Y167 | 3.07 | 0.89 | 1.12 | 0.96 | 0.26 | -0.14 |
| 591 | Y171 | 7.94 | 1.03 | 2.26 | 2.28 | 1.19 | 0.01 |
| 592 | Y171-1 | 3.80 | 0.76 | 1.06 | 1.07 | 0.40 | 0.01 |
| 593 | Y171-2 | 0.80 | 0.83 | 0.60 | 0.43 | -0.28 | -0.29 |
| 594 | Y172 1 | 3.00 | 0.74 | 1.07 | 1.03 | 0.45 | -0.03 |
| 595 | Y173 | 5.20 | 0.99 | 1.82 | 1.65 | 0.84 | -0.09 |
| 596 | Y174 | 7.90 | 0.98 | 2.57 | 2.71 | 1.62 | 0.05 |
| 597 | Y174-1 | 6.10 | 0.72 | 1.68 | 2.00 | 1.33 | 0.19 |
| 598 | Y176W-1 | 5.00 | 0.69 | 1.25 | 1.41 | 0.82 | 0.12 |
| 599 | Y176W-3 | 3.00 | 0.84 | 1.02 | 0.94 | 0.21 | -0.08 |
| 600 | Y176W-4 | 2.00 | 0.60 | 0.74 | 0.74 | 0.23 | 0.00 |
| 601 | Y177W-1 | 1.80 | 0.39 | 0.51 | 0.59 | 0.32 | 0.16 |
| 602 | Y2003 | 48.99 | 0.00 | 0.00 | 0.00 | 12.42 | 1.58 |
| 603 | Y2003-1 | 7.00 | 0.83 | 2.23 | 2.57 | 1.68 | 0.16 |
| 604 | Y222 | 2.15 | 0.00 | 0.00 | 0.00 | 0.56 | 0.39 |
| 605 | Y310E-1 | 1.00 | 0.58 | 0.55 | 0.50 | -0.06 | -0.08 |
| 606 | Y311-1 | 8.00 | 3.23 | 2.25 | -2.58 | -0.30 | -2.15 |
| 607 | Y311-2 | 2.00 | 0.28 | 0.37 | 0.43 | 0.29 | 0.16 |
| 608 | Y337 | 2.36 | 0.76 | 0.89 | 0.80 | 0.17 | -0.10 |
| 609 | Y337-1 | 0.30 | 0.75 | 0.45 | 0.27 | -0.41 | -0.39 |
| 610 | Y337S | 0.20 | 0.27 | 0.26 | 0.26 | -0.03 | -0.02 |
| 611 | Y337S-1 | 0.20 | 0.00 | 0.00 | 0.00 | 0.16 | 0.17 |
| 612 | Y337S-2 | 0.60 | 0.57 | 0.45 | 0.37 | -0.21 | -0.19 |
| 613 | Y337S-4 | 0.20 | 0.71 | 0.38 | 0.21 | -0.47 | -0.45 |
| 614 | Y339-1 | 4.00 | 0.26 | 0.52 | 0.72 | 0.96 | 0.40 |
| 615 | Y339-2 | 1.00 | 0.18 | 0.23 | 0.29 | 0.27 | 0.23 |
| 616 | Y339W-1 | 1.00 | 0.30 | 0.33 | 0.36 | 0.10 | 0.08 |
| 617 | Y342 | 0.30 | 0.79 | 0.52 | 0.35 | -0.34 | -0.32 |
| 618 | Y342-2 | 0.80 | 0.55 | 0.51 | 0.47 | -0.07 | -0.08 |
| 619 | Y348 | 0.20 | 0.23 | 0.23 | 0.23 | -0.01 | 0.00 |
| 620 | YP4 | 28.90 | 3.36 | 22.63 | -7.89 | 5.74 | -1.35 |
| 621 | S215-1 | 4.52 | 0.87 | 1.26 | 1.23 | 0.44 | -0.02 |
| 622 | S216-1 | 2.01 | 0.22 | 0.33 | 0.42 | 0.47 | 0.28 |
| 623 | S216-2 | 2.90 | 0.79 | 1.00 | 0.93 | 0.27 | -0.07 |
| 624 | S216E-1 | 1.31 | 0.31 | 0.37 | 0.42 | 0.21 | 0.14 |
| 625 | S251E-2 | 17.00 | 0.00 | 0.00 | 0.00 | 5.48 | 1.29 |
| 626 | S251E-3 | 1.50 | 0.00 | 0.00 | 0.00 | 0.42 | 0.34 |
| 627 | S253E-2 | 2.50 | 1.53 | 0.98 | 0.38 | -0.36 | -0.61 |
| 628 | S253E-3 | 2.36 | 0.87 | 0.92 | 0.79 | 0.06 | -0.15 |
| 629 | S263-2 | 3.00 | 2.09 | 1.05 | 0.25 | -0.50 | -0.76 |
| 630 | Y269E-2 | 1.10 | 1.25 | 0.46 | 0.15 | -0.63 | -0.68 |
| 631 | Y325S-1 | 2.00 | 0.11 | 0.19 | 0.27 | 0.69 | 0.46 |
| 632 | Y325S-2 | 1.00 | 0.00 | 0.00 | 0.00 | 0.26 | 0.24 |
| 633 | Y2151 | 3.11 | 1.36 | 1.26 | 0.83 | -0.07 | -0.34 |
| 634 | S228-2 | 3.65 | 0.42 | 0.65 | 0.78 | 0.57 | 0.20 |
| 635 | S228-3 | 6.32 | 0.48 | 1.23 | 1.71 | 1.56 | 0.40 |
| 636 | S229W-3 | 7.00 | 1.06 | 2.63 | 2.47 | 1.49 | -0.06 |
| 637 | S251-1 | 2.00 | 2.56 | -1.09 | 0.69 | -1.43 | -1.63 |
| 638 | S251-2 | 10.00 | 2.24 | 4.33 | 1.12 | 0.93 | -0.74 |
| 639 | S251-3 | 3.00 | 0.95 | 1.18 | 0.89 | 0.24 | -0.24 |
| 640 | S251-4 | 6.00 | 0.63 | 1.41 | 1.74 | 1.24 | 0.24 |
| 641 | Y216 | 12.40 | 1.02 | 3.55 | 4.16 | 2.49 | 0.17 |
| 642 | Y216-1 | 0.30 | 0.06 | 0.07 | 0.09 | 0.15 | 0.17 |
| 643 | Y216E-1 | 4.00 | 0.42 | 0.85 | 1.15 | 1.04 | 0.35 |
| 644 | Y216E-2 | 1.00 | 0.48 | 0.49 | 0.48 | 0.01 | -0.02 |
| 645 | Y218-1 | 3.00 | 0.88 | 1.14 | 0.95 | 0.30 | -0.16 |
| 646 | Y218-2 | 6.00 | 0.73 | 1.62 | 1.89 | 1.22 | 0.16 |
| 647 | Y251S-2 | 2.00 | 1.76 | 0.31 | -0.01 | -0.82 | -1.04 |
| 648 | Y271-2 | 0.50 | 0.22 | 0.24 | 0.26 | 0.08 | 0.09 |
| 649 | Y271S-1 | 3.00 | 0.21 | 0.36 | 0.48 | 0.68 | 0.34 |
| 650 | Y271S-2 | 4.50 | 1.11 | 1.77 | 1.28 | 0.59 | -0.28 |
| 651 | Y300 | 11.00 | 1.69 | 4.51 | 2.85 | 1.66 | -0.37 |
| 652 | Y300E-2 | 0.10 | 0.16 | 0.16 | 0.17 | 0.02 | 0.01 |
| 653 | Y300E-3 | 24.60 | 0.00 | 0.00 | 0.00 | 4.48 | 0.85 |
| 654 | Y331-1 | 8.00 | 0.97 | 2.75 | 2.96 | 1.84 | 0.07 |
| 655 | Y331-2 | 2.00 | 0.86 | 0.85 | 0.65 | -0.02 | -0.23 |
| 656 | Y331W-1 | 1.00 | 1.01 | 0.67 | 0.43 | -0.33 | -0.36 |
| 657 | Y331W-3 | 1.00 | 0.95 | 0.57 | 0.32 | -0.40 | -0.43 |
| 658 | Y333N-2 | 2.00 | 0.35 | 0.49 | 0.59 | 0.40 | 0.20 |
| 659 | C39 | 4.80 | 0.44 | 1.03 | 1.44 | 1.33 | 0.40 |
| 660 | Y122 | 1.27 | 1.19 | 0.67 | 0.33 | -0.44 | -0.50 |
| 661 | Y138 | 0.56 | 1.06 | 0.31 | 0.09 | -0.71 | -0.70 |
